# Piceatannol enhances antioxidant capacity and growth in weaned piglets by regulating of Nrf2-mediated redox homeostasis and modulating of the related gut microbiota

**DOI:** 10.1186/s40104-025-01320-8

**Published:** 2026-02-01

**Authors:** Longlong Zhu, Yuyan Che, Meixia Chen, Long Cai, Qiujue Wu, Tao Feng, Jing Wang

**Affiliations:** 1 Institute of Animal Husbandry and Veterinary Medicine (IAHVM), Beijing Academy of Agriculture and Forestry Sciences (BAAFS), Beijing, China; 2 College of Animal Science and Technology, Henan University of Science and Technology, Luoyang, China; 3 College of Animal Science and Technology, Gansu Agricultural University, Lanzhou, China; 4 Joint Laboratory of Animal Science between IAHVM of BAAFS and Division of Agricultural Science and Natural Resource of Oklahoma State University, Beijing, China

**Keywords:** Antioxidant, Growth performance, Intestinal flora, Nrf2 pathway, Piceatannol, Weaning piglets

## Abstract

**Background:**

Piglets are highly susceptible to oxidative stress, which can reduce growth performance and cause intestinal damage. Piceatannol (PIC), a natural bioactive substance enriched in Chinese rhubarb (*Rheum officinale*) and certain dark purple fruits, shows excellent antioxidant properties in our previous cell-based high-throughput screening. However, its effect on piglet growth performance and antioxidant capacity as well as underling mechanism has not been thoroughly investigated.

**Methods:**

One hundred weaned pigs (28 days of age, 8.71 ± 0.20 kg) were randomly assigned to 4 treatments with 5 replicates of 5 pigs per replicate. The experimental diets consisted of: 1) basal diet, 2) basal diet + 100 mg/kg PIC, 3) basal diet + 200 mg/kg PIC, and 4) basal diet + 300 mg/kg PIC. On d 15 and 35, one pig from each replicate was selected for sampling. The growth performance was monitored during a 35-day trial. In addition, H_2_O_2_-challenged IPEC-J2 cells served as an in vitro model to investigate the antioxidant mechanisms of PIC. IPEC-J2 cells were treated with 1,000 μmol/L H_2_O_2_ in the presence or absence of 10 μmol/L PIC.

**Results:**

Dietary PIC at 200 mg/kg significantly enhanced growth performance, as evidenced by increased average daily gain and feed conversion rate (*P* < 0.05). PIC supplementation markedly improved systemic antioxidant capacity, with elevated serum total antioxidant capacity, catalase activity, and glutathione levels, along with reduced malondialdehyde content (*P* < 0.05). Notably, PIC modulated the gut microbiota composition, increasing the amounts of beneficial genera (e.g., *Blautia* and *Faecalibacterium*), and these microbial shifts significantly correlated with improved antioxidant indices. In vitro, PIC pretreatment effectively protected IPEC-J2 cells against H_2_O_2_-induced oxidative damage by reducing reactive oxygen species generation and lipid peroxidation (*P* < 0.01). Mechanistically, PIC exerts its antioxidant effects through Nrf2 pathway activation, upregulating endogenous antioxidant enzymes (*P* < 0.05) while simultaneously inhibiting apoptosis via the regulation of the Bcl-2/Bax ratio and caspase-3 cleavage (*P* < 0.01).

**Conclusions:**

PIC improved the growth performance and health status of weaned piglets through the regulation of Nrf2-mediated redox homeostasis and modulation of the related gut microbiota, offering a potential new natural antioxidants for mitigating weaning stress in piglets.

**Graphical Abstract:**

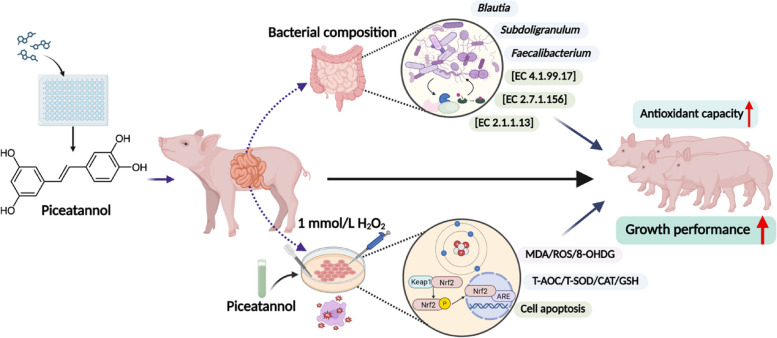

**Supplementary Information:**

The online version contains supplementary material available at 10.1186/s40104-025-01320-8.

## Introduction

In the pig industry, oxidative stress often leads to impaired growth performance and various diseases, resulting in significant economic losses [1]. Young animals, including piglets, have immature antioxidant defense systems, rendering them particularly susceptible to free radical accumulation, lipid peroxidation propagation, and consequent oxidative stress [2]. In intensive pig production, piglets are highly susceptible to oxidative stress caused by multiple stressors (e.g., environmental, nutritional, and psychological) [3], leading to suppressed growth performance and health status. Previous studies have demonstrated that early weaned piglets exhibit obvious oxidative stress, as evidenced by significantly elevated reactive oxygen species (ROS) and malondialdehyde (MDA) in the jejunum and liver, hydroxyl radicals in the colon, and hydrogen peroxide (H_2_O_2_) in the jejunum, colon, and liver [4, 5]. Continuous oxidative stress in piglets can damage cell function and organs and reduce production performance. Moreover, oxidative stress is an early event in the onset and development of various diseases and is therefore considered an important factor in early-weaning syndrome in piglets. In this context, the use of antioxidant feed additives has been proposed as an effective nutritional strategy to improve piglet health and growth performance.

Natural plant compounds and traditional Chinese medicine extracts have been widely investigated for their antioxidant properties [6]. Compounds such as carvacrol, thymol, quercetin, catechins, curcumin, oregano extract, and cinnamaldehyde have been demonstrated to function as effective antioxidants in livestock. These substances enhance the body’s antioxidant capacity and alleviate oxidative stress, thereby improving animal health and production performance [6–9]. We previously established a cell-based high-throughput screening assay to identify potential antioxidants from a traditional Chinese herbal compound library comprising 845 active monomers [10]. Piceatannol (PIC) was among the top 22 hits in a primary screening. In a dose–response-based secondary screening, 10 μmol/L PIC pretreatment maintained viability in H_2_O_2_-induced cells at 81.7%, compared to approximately 50% in non-pretreated cells [10]. PIC, a plant polyphenolic active substance, primarily derived from grapes, rhubarb, and sugarcane, has attracted considerable research interest due to its strong antioxidant properties [11, 12]. In C2C12 myotubes, PIC upregulated the expression of *HO1* and *SOD1*, thereby reducing H_2_O_2_-induced intracellular ROS accumulation [13]. In mice, PIC supplementation mitigated lipopolysaccharide-induced oxidative stress in liver tissues [14]. Nevertheless, literature on the effect of PIC supplementation on piglet growth performance and antioxidant capacity is limited.

Therefore, in the present study, we evaluated the effects of dietary PIC supplementation on growth performance and antioxidant capacity in weaning piglet and explored the underlying mechanisms of its antioxidant effects using a porcine intestinal epithelial cell model. We expect that our results may deepen the understanding of PIC’s antioxidant effects and mechanisms in piglets, thus providing a theoretical foundation for the development of effective antioxidants to alleviate oxidative stress in animals.

## Materials and methods

### Animal ethics statement

The animal experiments were conducted in compliance with the ethical guidelines approved by the Laboratory Animal Ethical Commission of the Institute of Animal Husbandry and Veterinary Medicine (IAHVM), Beijing Academy of Agriculture and Forestry Sciences (BAAFS; Beijing, China; approval No. IAHVM11-2304-21).

### Animal experimental design and management

A total of 100 piglets (Duroc × Landrace; 28 d old; 8.71 ± 1.36 kg) were selected and randomly assigned to four treatment groups. Each treatment group included five replicates, with five piglets per replicate. Piglets in the control group were administered a basal diet, whereas piglets in the treatment groups received the basal diet supplemented with 100 (low dose), 200 (medium dose), or 300 (high dose) mg/kg PIC (Great Forest Biomedical, Hangzhou, China; purity > 99%), respectively. PIC has the chemical formula C_14_H_12_O_4_, a molecular weight of 244.24, a CAS No. of 10083-24-6, and the following structural formula:  . The detailed composition of the basal diet, formulated according to the NRC (2012) and the Nutrient Requirements of Swine (2020) [15, 16], is presented in Table 1. The doses of PIC used in this experiment were based on previous reports on the structural analog, resveratrol [17, 18]. Ad libitum feeding and watering was maintained for all piglets during the entire experimental period. The formal experiment lasted 35 d. Body weight and feed intake were recorded for all piglets throughout the experimental period to determine growth performance parameters, including average daily gain (ADG), average daily feed intake (ADFI), and the feed conversion rate.

**Table 1:** Ingredients and nutrient composition of basal diets (as-fed basis), %

Items	d 0–15	d 15–35
Ingredients		
Corn	37.50	68.00
Extruded corn	11.00	^_^
Soybean meal	8.00	13.00
Extruded soybean	12.50	9.00
Wheat flour	18.50	^_^
Fish meal	5.10	3.50
Whey powder	4.00	3.70
Limestone	0.95	0.48
Dicalcium phosphate	0.11	0.70
Salt	0.30	0.30
L-Lysine HCl	0.58	0.32
DL-Methionine	0.13	^_^
L-Threonine	0.30	^_^
L-Tryptophan	0.03	^_^
Vitamin and mineral Premix^a^	1.00	1.00
Total	100.00	100.00
Nutrient levels^b^		
Digestible energy, MJ/kg	14.98	14.68
Crude protein	18.52	17.13
Calcium	0.80	0.70
Phosphorus	0.70	0.58
Lysine	1.40	1.17
Methionine	0.46	0.31
Threonine	0.84	0.55
Tryptophan	0.25	0.18

^a^ Each kg of ration provides: VA 9,800 IU; VB_1_ 4.5 mg; VB_2_ 11 mg; VB_6_ 6.5 mg; VB_12_ 0.1 mg; VD_3_ 2,700 IU; VE 50 IU; VK_3_ 4.1 mg; Folic acid 1.9 mg; Nicotinic acid 48 mg; D-pantothenate acid 25 mg; Biotin 0.3 mg; Cu 64 mg; Fe 100 mg; Mn 41 mg; Zn 100 mg; Se 0.3 mg; I 0.5 mg

^b^ Digestible energy is calculated according to NRC (2012) [15], other nutrient levels are analysed according to PRC National Standard [16]

### Serum sample collection and biochemical analysis

Following an overnight fast on d 15 and 35, blood samples (10 mL) were obtained from the anterior vena cava of each piglet. Serum was obtained by centrifugation at 4,000 × *g* for 10 min at 4 °C. Serum levels of total antioxidant capacity (T-AOC), glutathione (GSH), and MDA, along with activities of total superoxide dismutase (T-SOD) and catalase (CAT), were determined using commercial kits according to the manufacturer’s instructions. T-AOC, CAT, and GSH kits were obtained from Nanjing Jiancheng Bioengineering Institute (Nanjing, China), T-SOD and MDA kits from Beyotime Biotechnology (Shanghai, China). For data normalization, total protein concentrations were quantified using a bicinchoninic acid (BCA) assay (Huaxing bio, Beijing, China).

### 16S microbiome sequencing and bioinformatics analysis

On d 15 of the trial, fresh fecal samples (4–5 g) were collected from two or three piglets in each replicate per treatment and pooled together. The samples were immediately stored at −80 °C pending 16S rRNA sequencing for microbiota analysis. Total microbial genomic DNA was isolated from fecal samples using the E.Z.N.A. Stool DNA Kit (Omega Bio-Tek, Norcross, GA, USA) following the manufacturer’s protocol. The V3–V4 hypervariable regions of bacterial 16S rRNA genes were amplified with barcoded primers 338 F (5′-ACTCCTACGGGAGGCAGCAG-3′) and 806R (5′-GGACTACHVGGGTWTCTAAT-3′) in a T100 Thermal Cycler (Bio-Rad, Hercules, CA, USA). The PCR amplicons were size-fractionated by 2% agarose gel electrophoresis, purified using a PCR Clean-Up Kit (YuHua, Shanghai, China), and quantified with a Qubit 4.0 Fluorometer (Thermo Fisher Scientific, Waltham, MA, USA) employing the dsDNA HS Assay Kit. Equimolar amounts of purified amplicons were pooled for constructing sequencing libraries, which were paired-end sequenced (2 × 250 bp) on an Illumina NextSeq 2000 platform (Illumina, San Diego, CA, USA) at Majorbio Bio-Pharm Technology (Shanghai, China) following standard protocols. Following demultiplexing, raw sequences were processed through quality filtering using fastp (version 0.19.6) and merged with FLASH (v1.2.11). The resulting high-quality sequences were then subjected to denoising via the DADA2 plugin within the QIIME 2 pipeline (using default parameters), which provides single-nucleotide resolution by modeling sample-specific error profiles to generate amplicon sequence variants (ASVs). Taxonomic classification of ASVs was performed using the naive Bayes consensus taxonomy classifier implemented in QIIME 2, with reference to the SILVA 16S rRNA database. Metagenomic functions were predicted based on ASV representative sequences, using PICRUSt2 (Phylogenetic Investigation of Communities by Reconstruction of Unobserved States).

### Cell culture

IPEC-J2 porcine intestinal epithelial cells were generously provided by Dr. Glenn Zhang (Oklahoma State University, Stillwater, OK, USA). Cells were cultured in Dulbecco’s modified Eagle’s medium/Nutrient Mixture F-12 (Gibco/Thermo Fisher Scientific) supplemented with 10% (v/v) fetal bovine serum (Gibco), 100 μg/mL streptomycin, 100 U/mL penicillin, and 1% ITS supplement (5 μg/mL insulin, 5 μg/mL transferrin, 5 ng/mL selenium; ScienCell, San Diego, CA, USA) at 37 °C in a humidified 5% CO_2_ atmosphere. Routine subculturing was performed every 2–3 d.

### Oxidative stress model establishment and PIC treatment

An in vitro oxidative stress model was established by treating IPEC-J2 cells with H_2_O_2_. Cells were seeded in 96-well plates (Costar, Corning, NY, USA) at 8,000 cells/well (100 μL complete medium/well) and cultured for 40 h. Then, the cells were exposed to H_2_O_2_ (0–1,400 μmol/L in 100 μmol/L increments) for 4 h. Cell viability was assessed using a Cell Counting Kit-8 (CCK-8; Dojindo, Kumamoto, Japan) according to the manufacturer’s instructions. Absorbance was measured at 450 nm using a Multiskan FC instrument (Thermo Fisher Scientific).

Given that 4-h treatment with 1,000 μmol/L H_2_O_2_ reduced cell viability by ~ 50%, these parameter settings were employed in subsequent experiments. Cells were pretreated with PIC (0, 2.5, 5, 10, 20, or 40 μmol/L) for 24 h and then exposed to 1,000 μmol/L H_2_O_2_ for 4 h prior to the CCK-8 assay. Non-treated cells served as controls. Pretreatment with 10 μmol/L PIC conferred the strongest protective effect against H_2_O_2_-induced damage in terms of cell viability. Morphological changes in IPEC-J2 cells were examined using an optical microscope (Olympus, Tokyo, Japan; 100 × magnification), and images were captured using a cellSens Entry system (Olympus). Culture supernatants were centrifuged (400 × *g*, 10 min), and lactate dehydrogenase (LDH) activity was measured using a Cytotoxicity Detection Assay Kit (C0016, Beyotime Biotechnology) on an Infinite 200 PRO microplate reader (Tecan Trading AG, Switzerland).

### Determination of MDA production, ROS accumulation, and antioxidant activities

IPEC-J2 cells were seeded in 6-well plates (2.5 × 10^5^ cells/well) and cultured overnight. Cells were pretreated or not with 10 μmol/L PIC for 24 h and then exposed or not to 1,000 μmol/L H_2_O_2_ for 4 h. Post-treatment, the cells were lysed in radioimmunoprecipitation assay (RIPA) buffer (containing phenylmethylsulfonyl fluoride; Solarbio, Beijing, China) for 15 min. The lysates were centrifuged (12,000 × *g*, 4 °C, 15 min), and the supernatants were collected for analysis. Proteins were quantified using a BCA assay kit.

The concentration of MDA was determined using a Lipid Peroxidation MDA Assay Kit (S0131S; Beyotime Biotechnology) following the manufacturer’s instructions. Supernatants after incubation were collected to determine the 8-hydroxy-2′-deoxyguanine (8-OHDG) levels (Jiangsu Meibiao Biotechnology, Jiangsu, China). For ROS detection, cells were trypsinized, washed twice, stained with dichlorodihydrofluorescein diacetate probe (E004-1-1; Nanjing Jiancheng Bioengineering Institute) at 37 °C for 1 h, and analyzed using an Infinite 200 PRO microplate reader (Tecan Trading AG). T-AOC, T-SOD, and CAT activities and GSH levels in cell lysates were measured using the above-mentioned commercial kits.

### Quantitative reverse transcription (RT-qPCR) analysis

Following cell treatments, total RNA was isolated using RNAzol (Molecular Research Center, Cincinnati, OH, USA) and alcohol precipitation. RNA concentration and quality were measured using a DS-11 spectrophotometer (DeNovix, Wilmington, DE, USA). First-strand cDNA was synthesized from 1 μg RNA using an iScript cDNA synthesis kit (Bio-Rad, Hercules, CA, USA) following the manufacturer’s protocol. qPCRs were run using iTaq Universal SYBR Green Supermix (Bio-Rad) on a QuantStudio 3 system (Thermo Fisher Scientific) with porcine-specific primers (Table S1). The thermal cycling protocol consisted of 40 cycles of 95 °C for 30 s (denaturation), 60 °C for 30 s (annealing), and 72 °C for 20 s (extension). Target gene expression levels were normalized to *GAPDH* expression and calculated using the 2^−ΔΔCT^ method [19].

### Western blot analysis

Following cell treatments, proteins were extracted with RIPA lysis buffer, and their concentrations were quantified by BCA assay followed by normalization. Samples were denatured in 5 × SDS loading buffer (Beyotime Biotechnology) at 95 °C for 5 min and resolved by 10%−12% SDS polyacrylamide gel electrophoresis. Following electrophoretic separation, proteins were transferred onto polyvinylidene difluoride membranes. The membranes were then blocked with 5% (w/v) skim milk in Tris-buffered saline containing Tween-20 for 3 h at room temperature, followed by overnight incubation with primary antibodies at 4 °C overnight. After washing, the membranes were probed with horseradish peroxidase-conjugated secondary antibodies for 1 h. All antibodies used in this study are listed in Table S2. Immunoreactive bands were visualized using an ECL-Plus chemiluminescence substrate (Beyotime Biotechnology) and imaged with a ChemiDoc Imaging System (Bio-Rad Laboratories). Band intensities were quantified using Image J software (National Institutes of Health, Bethesda, MD, USA).

### Establishment of *Nrf2*-knockdown IPEC-J2 cells

Porcine *Nrf2* siRNA (sense strand: 5′-GCCCAUUGAUCUCUCUGAUTT-3′; antisense strand: 5′-AUCAGAGAGAUCAAUGGGCTT-3′), synthesized at GenePharma (Shanghai, China), was transfected into IPEC-J2 cells using Lipofectamine 3000 (Invitrogen, Carlsbad, CA, USA) following the manufacturer’s protocol. Successfully transfected cells were pretreated or not with 10 μmol/L PIC for 24 h and then exposed to 1,000 μmol/L H_2_O_2_ for 4 h. Non-siRNA-treated cells served as controls. Cell viability, MDA concentration, and ROS accumulation were measured as described above.

### Statistical analysis

In vivo and in vitro experimental data were processed using GraphPad Prism v6 (GraphPad Software, San Diego, CA, USA). All data are presented as mean and standard error of the mean (SEM) and statistically analyzed using the mixed linear model in SPSS (v20.0, SPSS, Chicago, IL, USA) as follows:

$$Y_{ijk}=\mu+T_i+P_j+S_k+e_{ijk},$$

where *Y*_*ijk*_ is the dependent variable; *μ* is the overall mean; *T*_*i*_ is the fixed treatment effect, *P*_*j*_ is the period effect; *S*_*k*_ is the steer effect; *e*_*ijk*_ is random error.

One-way analysis of variance (ANOVA) followed by Duncan’s multiple range test in SPSS was used to identify significant differences among the groups in piglet experiment. Linear and quadratic effects of dietary PIC supplementation doses were evaluated using regression analysis. An unpaired two-tailed Student’s *t*-test was used to compare group means in porcine cell experiments. Statistical significance was set to *P* < 0.05.

Gut microbiota bioinformatics analysis was conducted using the Majorbio Cloud platform (https://cloud.majorbio.com). Microbial community similarity among samples was evaluated through principal coordinate analysis (PCoA) based on Bray–Curtis dissimilarity matrices using the Vegan package (v2.5-3). Treatment effects on community composition were statistically analyzed by PERMANOVA, which quantified the percentage of variation explained and its statistical significance. Linear discriminant analysis effect size (LEfSe) analysis was conducted to identify taxa with significantly different abundances (from phylum to genus level) of bacteria among groups based on linear discriminant analysis score > 2.5 and *P* < 0.05. Significant correlations between nodes were defined as Spearman’s |ρ| > 0.6 and false discovery rate-adjusted *P* < 0.01.

## Results

### PIC improves growth performance

Dietary PIC supplementation improved weight gain in piglets (Table 2). On d 35 of the trial, the average weight in each treatment group was higher than that in the control group, with a significant difference observed in the medium-dose PIC group (*P* < 0.05). PIC supplementation increased the ADG during the d 0–15 and d 0–35 periods, with a significant effect in the medium-dose group (*P* < 0.05). During the d 0–15 period, the addition of medium and high doses of PIC significantly improved the ADFI of piglets (*P* < 0.05). The addition of PIC reduced the feed conversion efficiency during piglet rearing, and the feed-to-gain ratio was significantly lower in the medium-dose group compared with the control group (*P* < 0.05) over the entire d 0–35 period. Furthermore, with increasing PIC dose, the final weight of piglets on d 35 and the d 0–35 ADG showed linear and quadratic responses (*P* < 0.05). The final weight on d 15, d 0–15 ADG, and ADFI exhibited a linear increase (*P* < 0.05), whereas the d 0–35 feed-to-gain showed a linear decrease (*P* < 0.05).

**Table 2:** Effects of piceatannol (PIC) on growth performance in weaning piglets

Items	Piceatannol, mg/kg				SEM	*P*-value		
	0	100	200	300		ANOVA	Linear	Quadratic
BW, kg								
d 0	8.72	8.70	8.69	8.70	0.133	1.000	0.965	0.965
d 15	15.0	15.3	15.9	15.7	0.157	0.163	0.047	0.437
d 35	28.0^b^	29.2^ab^	29.9^a^	29.4^ab^	0.241	0.015	0.010	0.035
ADG, g								
d 0–15	418^b^	440^ab^	479^a^	469^ab^	8.796	0.039	0.011	0.285
d 15–35	649	693	703	682	11.51	0.397	0.296	0.178
d 0–35	550^b^	584^ab^	607^a^	591^ab^	7.135	0.020	0.012	0.042
ADFI, g								
d 0–15	717^b^	751^ab^	799^a^	791^a^	11.05	0.016	0.003	0.254
d 15–35	1,199	1,212	1,222	1,196	13.94	0.919	0.995	0.522
d 0–35	993	1,014	1,041	1,022	9.886	0.412	0.211	0.326
F/G								
d 0–15	1.74	1.71	1.67	1.69	0.029	0.874	0.519	0.653
d 15–35	1.86	1.75	1.74	1.75	0.024	0.264	0.127	0.217
d 0–35	1.81^a^	1.74^ab^	1.72^b^	1.73^ab^	0.012	0.028	0.015	0.053

Mean and total SEM are listed in separate columns (*n* = 5)

^a,b^Different superscript letters within a row indicate significant differences (*P* < 0.05)

*BW* Body weight, *ADG* Average daily gain, *ADFI* Average daily feed intake, *FCR* Feed conversion rate

### PIC improves serum antioxidant status

The effects of PIC on piglet antioxidant indicators are shown in Table 3. In comparison to the control group, the low-dose PIC group had significantly enhanced serum T-AOC on d 15 and CAT activity on d 35 (*P* < 0.05). The medium-dose PIC group showed more comprehensive effects, with significantly increased T-AOC and reduced MDA content on d 15 and 35, along with elevated CAT activity on d 35 (*P* < 0.05). The high-dose PIC group exhibited distinct regulatory effects, with significantly reduced MDA contents on d 15 and increased GSH levels on d 35 (*P* < 0.05). PIC administration did not significantly affect T-SOD activity on d 15 or 35, nor CAT activity and GSH content on d 15 (*P* > 0.05). Dose–response analysis revealed that d 15 T-AOC levels responded to PIC supplementation in both linear (*P* < 0.05) and quadratic (*P* < 0.01) patterns. Linear relationships were observed for GSH levels on d 35, as well as d 15 MDA content (*P* < 0.01). Quadratic responses were identified for d 35 T-AOC levels (*P* < 0.01), CAT activity (*P* < 0.01), and MDA content (*P* < 0.05), indicating complex dose-dependent effects of PIC on the antioxidant system during different developmental stages.

**Table 3:** Effects of piceatannol (PIC) on serum antioxidant capacity in weaning piglets

Items	Piceatannol, mg/kg				SEM	*P*-value		
	0	100	200	300		ANOVA	Linear	Quadratic
d 15								
T-SOD, U/g protein	0.90	0.97	0.94	0.79	0.031	0.166	0.184	0.067
T-AOC, mmol/g protein	0.77^b^	0.91^a^	0.92^a^	0.87^ab^	0.018	0.006	0.023	0.004
CAT, U/mL	1.65	1.94	2.52	2.19	0.178	0.390	0.190	0.394
GSH, μmol/g protein	10.32	9.19	10.50	9.30	0.247	0.121	0.400	0.934
MDA, μmol/mg protein	3.63^a^	3.28^ab^	2.33^b^	2.52^b^	0.174	0.010	0.003	0.332
d 35								
T-SOD, U/g protein	0.85	1.17	0.89	0.82	0.074	0.321	0.545	0.199
T-AOC, mmol/g protein	0.19^b^	0.22^ab^	0.23^a^	0.20^ab^	0.006	0.007	0.408	0.001
CAT, U/mL	1.50^b^	2.15^a^	2.12^a^	1.80^ab^	0.083	0.006	0.139	0.001
GSH, μmol/g protein	12.41^b^	13.35^ab^	13.62^ab^	13.92^a^	0.190	0.015	0.003	0.308
MDA, μmol/mg protein	7.68^a^	6.16^ab^	4.54^b^	6.68^ab^	0.427	0.046	0.162	0.021

Mean and total SEM are listed in separate columns (*n* = 5)

^a,b^Different superscript letters within a row indicate significant differences (*P* < 0.05)

*T-SOD* Total superoxide dismutase, *T-AOC* Total antioxidant capacity, *CAT* Catalase, *GSH* Glutathione, *MDA* Malondialdehyde

### Dietary PIC supplementation alters gut microbial diversity and structure in weanling piglets

**Fig. 1:**
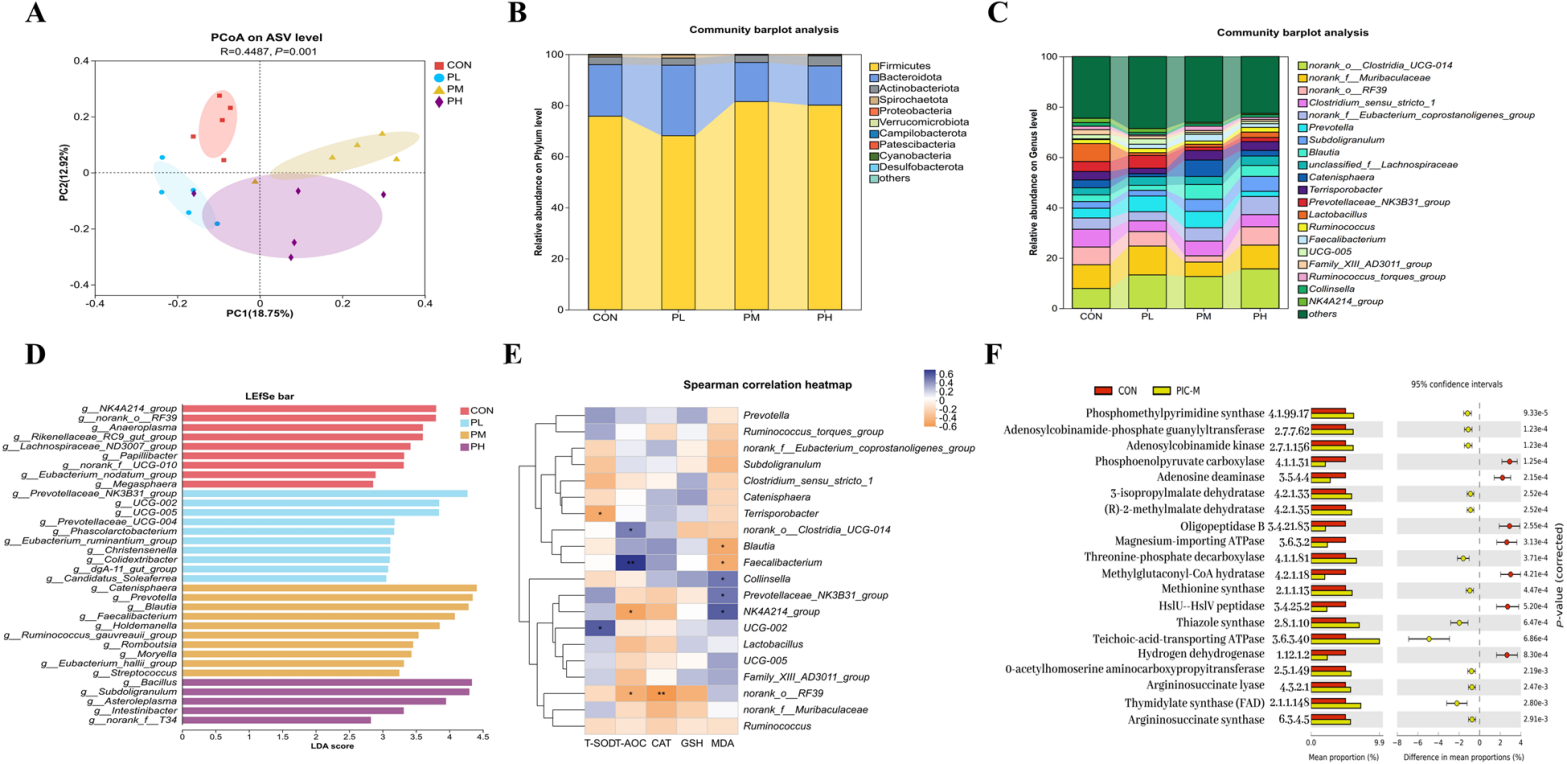
16S rRNA microbiome sequencing in weaning piglets on PIC supplementation, and bioinformatics analysis. **A** Principal coordinate analysis (PCoA) plot generated based on an unweighted UniFrac matrix. **B** Phylum-level microbiota profiles of PIC-treated and non-treated control piglets. **C** Genus-level microbiota profiles of PIC-treated and control piglets. **D** Differences between bacterial taxa in PIC-treated and control piglets. The histogram shows differentially abundant bacterial species in the treatment and control groups ranked by LDA scores. **E** Spearman correlation heatmaps of the correlations between serum antioxidant indices and relative abundances of fecal bacterial genera. Only the top 20 bacterial genera are shown. Asterisks represent significant positive or negative correlations (^*^P < 0.05, ^**^P < 0.01). **F** Significant differences in PICRUSt2-predicted gene counts for enzymes between medium dose treatment (200 mg/kg PIC) and control groups. Only the top 20 enzymes in terms of significance are shown. *n* = 5

PIC supplementation significantly altered β-diversity in the gut microbiota of piglets as indicated by PCoA results (*P* < 0.01) (Fig. 1A). On d 15 post-weaning, the fecal microbiota composition was dominated by four major phyla: Firmicutes, Bacteroidota, Actinobacteriota, and Spirochaetota, which collectively accounted for > 99% of the total relative abundance (Fig. 1B). The dominant bacterial genera included *norank_o_Clostridia_UCG-014*, *norank_f_Muribaculaceae*, *norank_o_RF39*, *Clostridium_sensu_stricto_1*, *norank_f_Eubacterium_coprostanoligenes_group*, and *Prevotella* (Fig. 1C). LEfSe analysis revealed significant microbial enrichments across groups (Fig. 1D). Genera such as *Prevotellaceae_NK3B31_group*, *UCG-002*, and *UCG-005* were significantly enriched in the low-dose PIC group. *Catenisphaera*, *Prevotella*, and *Blautia*, were significantly enriched specifically in the medium-dose PIC group, whereas the high-dose PIC group was significantly enriched in *Bacillus*, *Subdoligranulum*, and *Asteroleplasma*.

### Dietary PIC supplementation attenuates oxidative stress in association with gut microbiota alterations in weaning piglets

The correlation heatmap based on correlation coefficients in Fig. 1E depicts the relationships between microbial species in piglet feces and serum antioxidant indices. T-AOC exhibited positive correlations with *norank_o__Clostridia_UCG-014* and *Faecalibacterium*, and negative correlations with *NK4A214_group* and *norank_o__RF39* (*P* < 0.05). Serum T-SOD activity was significantly positively correlated with *UCG-002*, and negatively with *Terrisporobacter* (*P* < 0.05). Serum CAT activity exhibited a significant negative correlation with *norank_o__RF39* (*P* < 0.05). Serum MDA content was significantly positively correlated with *Collinsella*, *NK4A214_group*, *Prevotellaceae_NK3B31_group* (*P* < 0.05), but negatively with *Blautia* and *Faecalibacterium* (*P* < 0.05).

KEGG functional prediction analysis of microbial communities using PICRUSt2 annotated a total of 1,770 enzymes. Figure 1F presents a heatmap of the top 20 most significantly enriched enzyme categories. Dietary supplementation with 200 mg/kg PIC significantly increased the abundances of phosphomethylpyrimidine synthase [EC 4.1.99.17], methionine synthase [EC 2.1.1.13], and adenosylcobinamide kinase [EC 2.7.1.156] in the piglet gut microbiota, whereas it significantly reduced the abundances of phosphoenolpyruvate carboxylase [EC 4.1.1.31], adenosine deaminase [EC 3.5.4.4], and oligopeptidase B [EC 3.4.21.83].

### PIC exerts a cytoprotective in IPEC-J2 cells

**Fig. 2:**
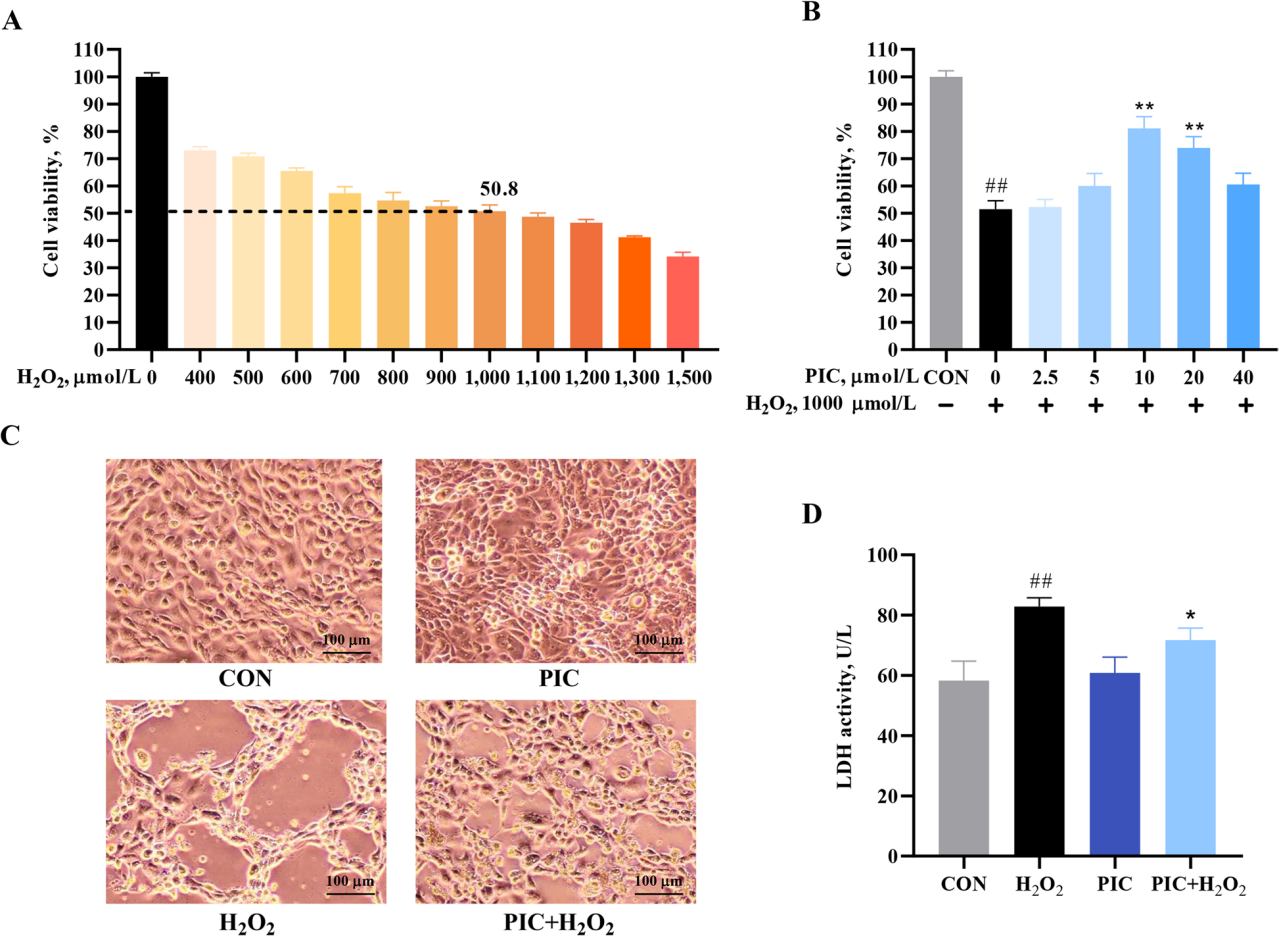
PIC ameliorates H_2_O_2_-induced damage in IPEC-J2 cells. **A** Cell viability of IPEC-J2 cells after treatment with H_2_O_2_ at different concentrations. **B** Protective effect of PIC on H_2_O_2_-induced cell damage at different concentrations. **C** Morphological analysis of the protective effect of PIC against H_2_O_2_-induced IPEC-J2 cell damage using microscopy. **D** Effect of PIC on lactate dehydrogenase (LDH) release. CON, cells not treated with H_2_O_2_ and PIC; H_2_O_2_, cells treated with 1,000 μmol/L H_2_O_2_ for 4 h; PIC, cells treated with 10 μmol/L PIC for 24 h; PIC + H_2_O_2_, cells pretreated with 10 μmol/L PIC for 24 h and then treated with 1,000 μmol/L H_2_O_2_ for 4 h. Data are presented as mean ± SEM (*n* = 6). ^#^*P* < 0.05 and ^##^*P* < 0.01 vs. control, ^*^P < 0.05 and ^**^P < 0.01 vs. H_2_O_2_

Cell viability was the highest in the (non-treated) control group, indicating normal cellular conditions. Exposure to H_2_O_2_ decreased cell viability in a concentration-dependent manner, indicating strong oxidative stress-induced cytotoxicity (Fig. 2A). PIC pretreatment improved viability compared to H_2_O_2_ alone, particularly at PIC concentrations of 10–20 μmol/L, suggesting a protective role of PIC against oxidative damage (Fig. 2B). Morphological analysis revealed that cells in the control group showed normal cell morphology, whereas H_2_O_2_-treated IPEC-J2 cells exhibited enlarged intercellular gaps and disrupted membrane integrity (Fig. 2C). PIC pretreatment significantly attenuated these morphological alterations and effectively preserved cellular integrity. LDH activity is a marker of cell membrane integrity. LDH levels were the lowest in the control group, consistent with minimal cellular damage (Fig. 2D). Cells in the H_2_O_2_-treated group displayed significantly elevated LDH activity (*P* < 0.01), indicating cellular membrane disruption. Cells in the PIC + H_2_O_2_ group showed reduced LDH release compared with those treated with H_2_O_2_ alone (*P* < 0.05), indicating a cytoprotective effect of PIC.

### Characterization of the antioxidant activities of PIC

**Fig. 3:**
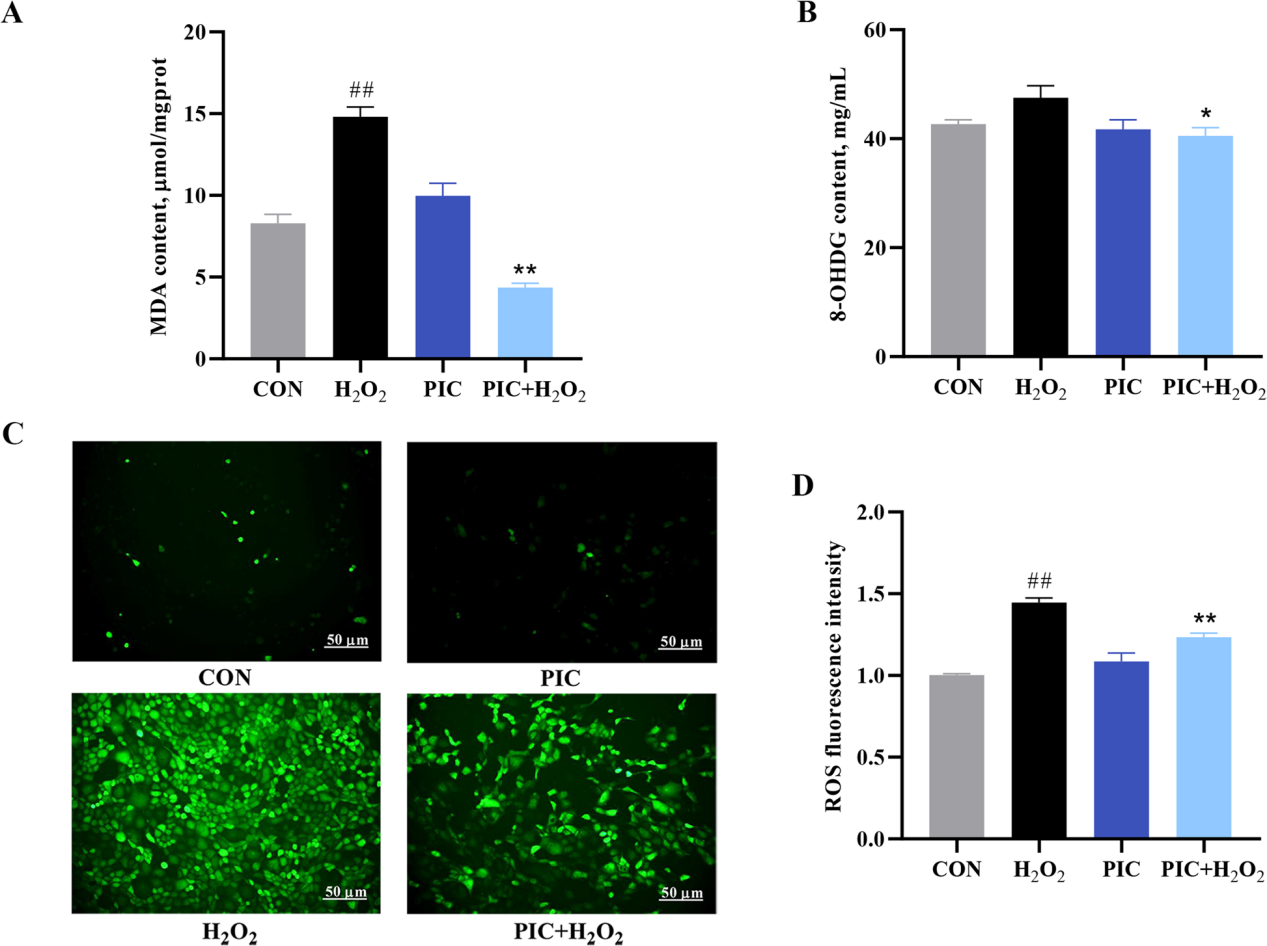
PIC attenuates redox disturbance in H_2_O_2_-induced IPEC-J2 cells. **A** Effect of PIC on H_2_O_2_-induced MDA production. **B** Effect of PIC on H_2_O_2_-induced 8-OHDG production. **C** Representative images of dichlorodihydrofluorescein diacetate staining. **D** Quantitation of ROS levels based on dichlorodihydrofluorescein diacetate fluorescence in panel C. CON, cells not treated with H_2_O_2_ and PIC; H_2_O_2_, cells treated with 1,000 μmol/L H_2_O_2_ for 4 h; PIC, cells treated with 10 μmol/L PIC for 24 h; PIC + H_2_O_2_, cells pretreated with 10 μmol/L PIC for 24 h and then treated with 1,000 μmol/L H_2_O_2_ for 4 h. Data are presented as mean ± SEM (*n* = 6). ^#^*P* < 0.05 and ^##^*P* < 0.01 vs. control, ^*^P < 0.05 and ^**^P < 0.01 vs. H_2_O_2_

Figure 3 illustrates the production of oxidative stress products, including MDA, 8-OHDG, and ROS, in IPEC-J2 cells in the different treatment groups. The control group exhibited the lowest MDA content. The H_2_O_2_-treated group displayed a significant increase in MDA levels, suggesting pronounced oxidative stress. Pretreatment with PIC significantly reduced the MDA content compared with H_2_O_2_ treatment alone (*P* < 0.01), implying that PIC effectively mitigates H_2_O_2_-induced lipid peroxidation. 8-OHDG is a major hallmark of DNA oxidative damage. PIC pretreatment significantly suppressed 8-OHDG production compared with H_2_O_2_ treatment alone (*P* < 0.05). ROS generation was measured based on fluorescence intensity. H_2_O_2_ treatment enhanced ROS levels by 1.45-fold compared to those in the control group, indicating oxidative stress induction. ROS levels were maintained at control levels in the PIC alone group, and the PIC + H_2_O_2_ group exhibited only partial ROS elevation compared to H_2_O_2_ alone group. These findings suggest that PIC prevents spontaneous ROS formation and partially counteracts H_2_O_2_-induced oxidative stress.

### Effects of PIC on cell apoptosis

**Fig. 4:**
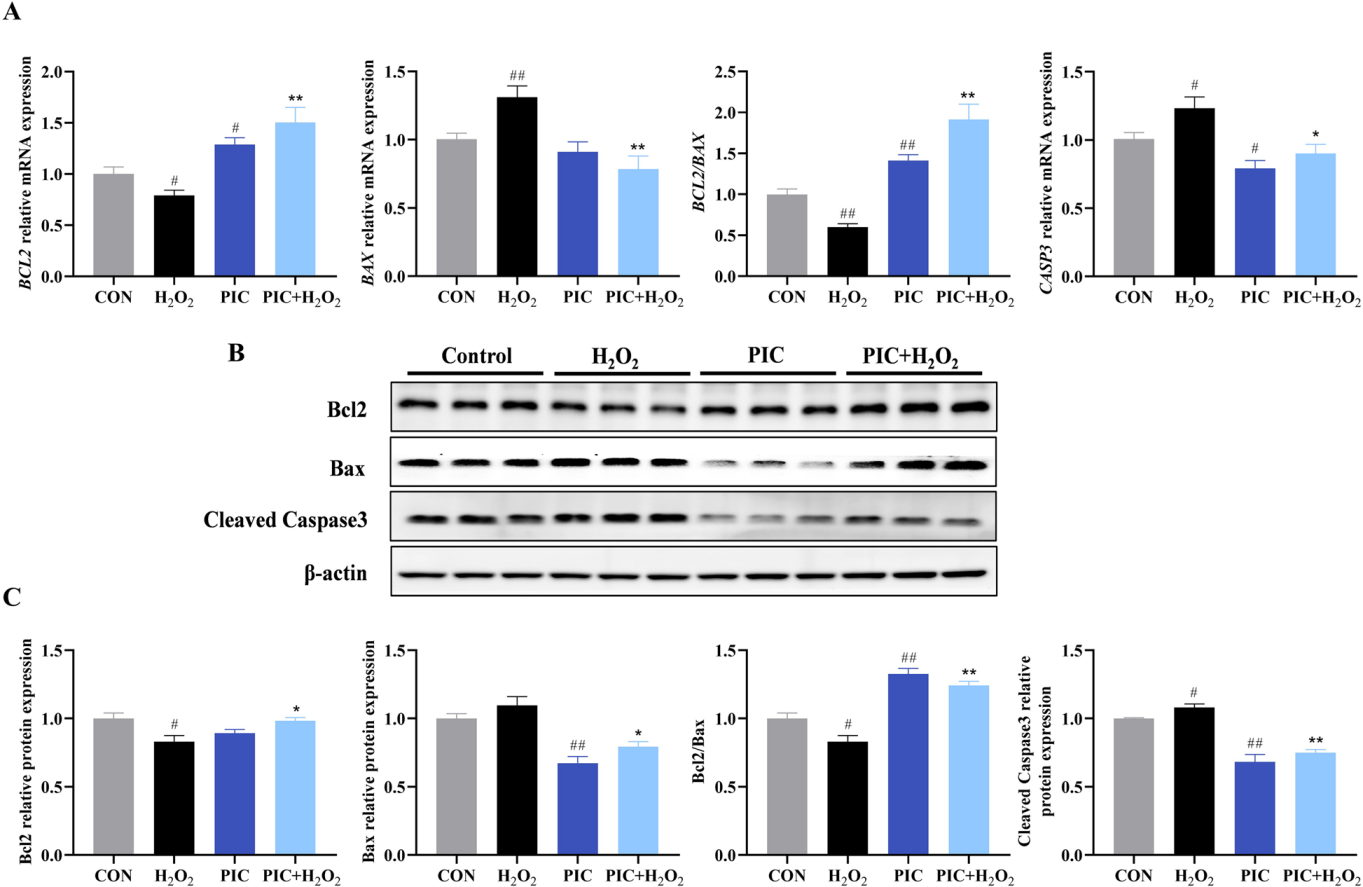
PIC attenuates apoptosis in H_2_O_2_-induced IPEC-J2 cells. **A** mRNA expression of apoptosis-associated genes measured by RT-qPCR. **B** Representative western blot image of apoptosis-related proteins. **C** Quantitative results of protein levels in panel **B**. CON, cells not treated with H_2_O_2_ and PIC; H_2_O_2_, cells treated with 1,000 μmol/L H_2_O_2_ for 4 h; PIC, cells treated with 10 μmol/L PIC for 24 h; PIC + H_2_O_2_, cells pretreated with 10 μmol/L PIC for 24 h and then treated with 1,000 μmol/L H_2_O_2_ for 4 h. are presented as mean ± SEM (*n* = 6). ^#^*P* < 0.05 and ^##^*P* < 0.01 vs. control, ^*^P < 0.05 and ^**^P < 0.01 vs. H_2_O_2_

Excess intracellular ROS trigger apoptosis. The effects of PIC on H_2_O_2_-induced apoptosis are shown in Fig. 4. RT-qPCR analysis revealed that H_2_O_2_ treatment markedly upregulated pro-apoptotic genes while downregulating anti-apoptotic genes. Notably, PIC alone significantly elevated B-cell lymphoma 2 (*BCL2*) expression and decreased the expression of Caspase 3 (*CASP3*) (*P* < 0.05). PIC pretreatment significantly alleviated H_2_O_2_-induced apoptosis as demonstrated by decreased Bcl-2-associated X protein (*BAX; P *< 0.01) and *CASP3* (*P* < 0.05) expression and increased *BCL2* (*P* < 0.01) expression compared to the levels after H_2_O_2_ treatment alone. Consistent with the trends observed in the RT-qPCR results, western blotting revealed that H_2_O_2_ treatment reduced Bcl-2 expression and enhanced Bax and Cleaved Caspase 3 expression, whereas PIC reversed these effects.

### Effects of PIC on the antioxidant defense system

**Fig. 5:**
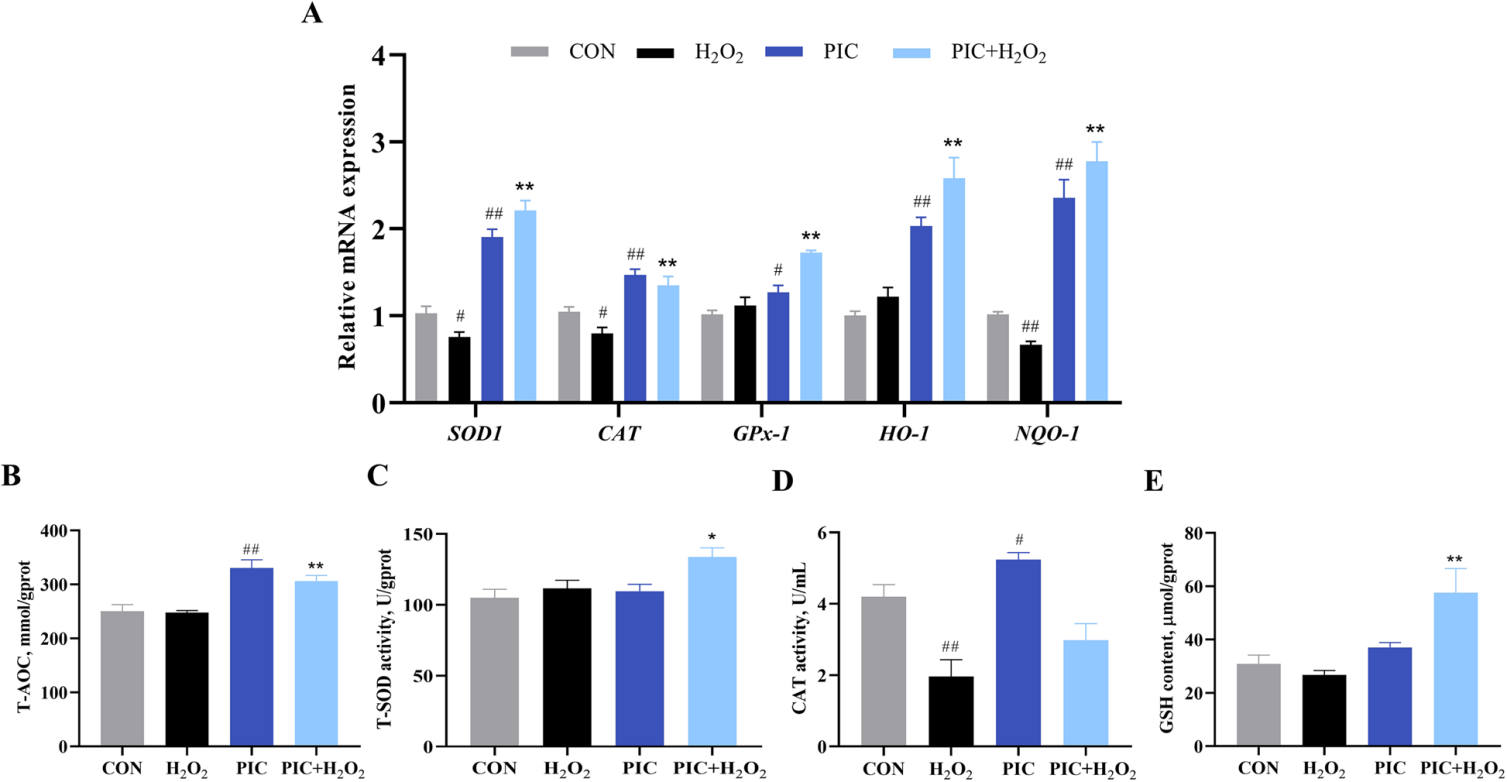
PIC improves antioxidant capacity in H_2_O_2_-induced IPEC-J2 cells. **A** mRNA expression of *SOD1*, *CAT*, *GPX1*, *HO1*, and *NQO1* measured by RT-qPCR. **B** Effect of PIC on H_2_O_2_-induced total antioxidant capacity. **C** Effect of PIC on H_2_O_2_-induced superoxide dismutase activity. **D** Effect of PIC on H_2_O_2_-induced catalase activity. **E** Effect of PIC on H_2_O_2_-induced reduced glutathione. CON, cells not treated with H_2_O_2_ and PIC; H_2_O_2_, cells treated with 1,000 μmol/L H_2_O_2_ for 4 h; PIC, cells treated with 10 μmol/L PIC for 24 h; PIC + H_2_O_2_, cells pretreated with 10 μmol/L PIC for 24 h and then treated with 1,000 μmol/L H_2_O_2_ for 4 h. are presented as mean ± SEM (*n* = 6). ^#^*P* < 0.05 and ^##^*P* < 0.01 vs. control, ^*^P < 0.05 and ^**^P < 0.01 vs. H_2_O_2_

As shown in Fig. 5A, PIC treatment alone significantly upregulated the mRNA expression of *SOD1*, *CAT*, *GPX1*, *HO1*, and *NQO1*, demonstrating its intrinsic antioxidant capacity. Compared with H_2_O_2_ treatment alone, PIC pretreatment significantly attenuated H_2_O_2_-induced alterations in these antioxidant enzyme transcript levels. Furthermore, PIC pretreatment markedly elevated T-AOC levels compared to H_2_O_2_ treatment alone (Fig. 5B). Consistent with these gene expression profiles, PIC pretreatment alleviated T-SOD, GSH, and CAT activities compared to H_2_O_2_ treatment alone, although the effect on CAT was not significant. These results suggest that PIC may confer protective effects by activating endogenous antioxidant defense systems.

### PIC antioxidant capacity involves Nrf2 signaling

**Fig. 6:**
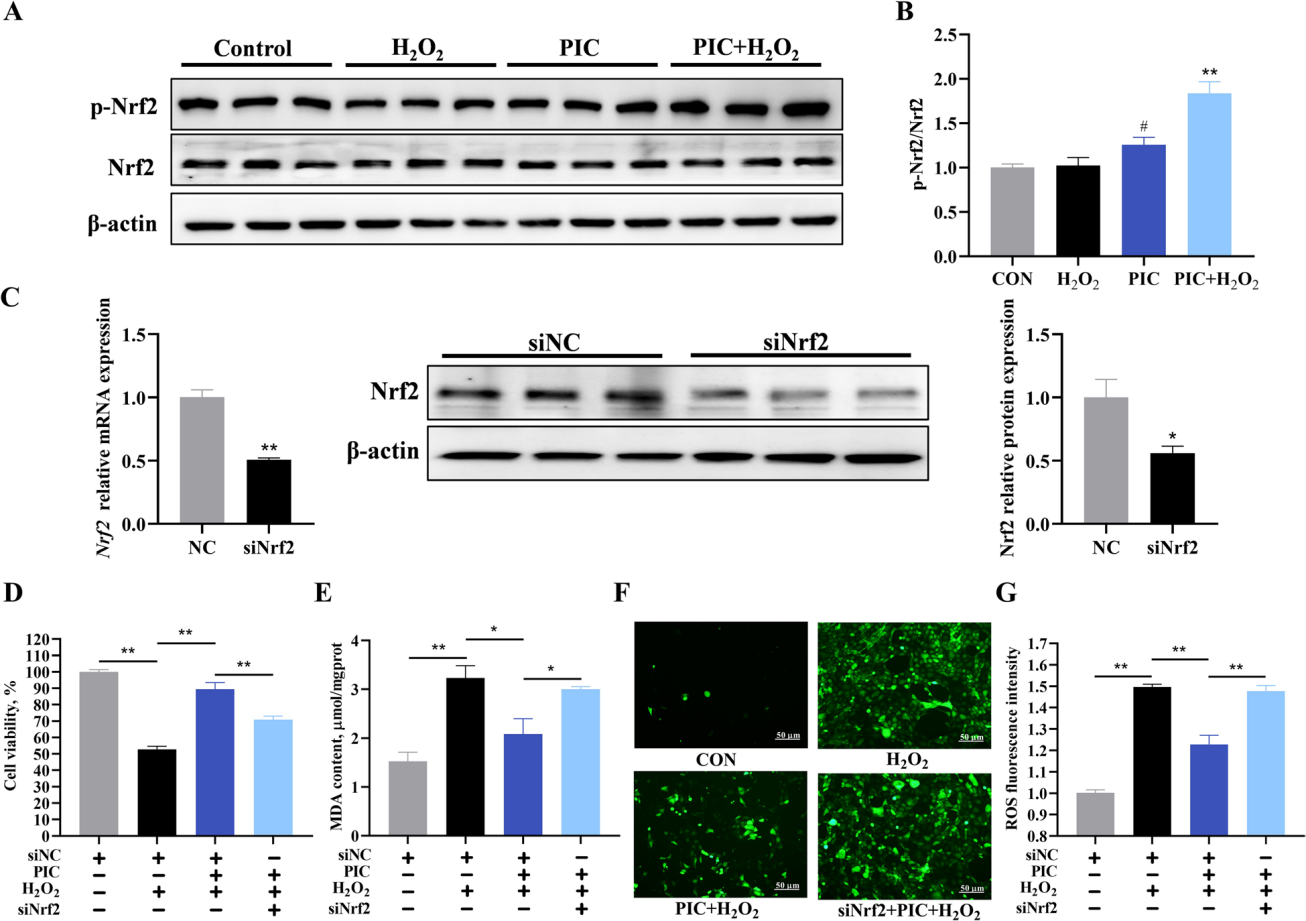
PIC alleviates oxidative stress via the Nrf2 pathway in H_2_O_2_-induced IPEC-J2 cells. **A** Western blots of Nrf2 and p-Nrf2 levels. **B** Quantitation of Nrf2 and p-Nrf2 levels in panel **A**. **C** mRNA and protein levels of Nrf2 after Nrf2 silencing. **D** Cell viability, normalized to that of negative control siRNA-transfected cells. **E** Effect of PIC on H_2_O_2_-induced MDA concentration in *Nrf2*-knockdown cells. **F** Effect of PIC on H_2_O_2_-induced ROS production in *Nrf2*-knockdown cells. **G** Quantitation of ROS levels in panel F. CON, cells not treated with H_2_O_2_ and PIC; H_2_O_2_, cells treated with 1,000 μmol/L H_2_O_2_ for 4 h; PIC, cells treated with 10 μmol/L PIC for 24 h; PIC + H_2_O_2_, cells pretreated with 10 μmol/L PIC for 24 h and then treated with 1,000 μmol/L H_2_O_2_ for 4 h. Data are presented as mean ± SEM (n = 6). ^#^*P* < 0.05 and ^##^*P* < 0.01 vs. control, ^*^P < 0.05 and ^**^P < 0.01 vs. H_2_O_2_. In *Nrf2*-knockdown cells, ^*^P < 0.05 and ^**^P < 0.01

To explore the protective mechanisms of PIC further, we assessed whether Nrf2 activation contributed to the enhanced antioxidant capacity observed in PIC-pretreated IPEC-J2 cells (Fig. 6). Immunoblotting results revealed that PIC pretreatment promoted Nrf2 accumulation, indicating Nrf2 pathway activation. To further elucidate the role of Nrf2 in PIC-mediated attenuation of oxidative stress, we generated *Nrf2*-knockdown IPEC-J2 cells and assessed treatment effects on cell viability, MDA generation, and ROS production. Nrf2 siRNA-transfected cells demonstrated significant downregulation of Nrf2 expression compared to negative control siRNA-treated cells, as confirmed by RT-qPCR and western blot analyses. Following PIC pretreatment and H_2_O_2_ challenge, *Nrf2*-knockdown IPEC-J2 cells exhibited significantly reduced viability compared to control siRNA-transfected cells (*P* < 0.01). *Nrf2* knockdown significantly weakened PIC’s preventing effect on H_2_O_2_-induced MDA (*P* < 0.05) and ROS accumulation (*P* < 0.01). These results suggest that the oxidation-protective effect of PIC was blocked by Nrf2 siRNA, indicating the involvement of Nrf2 signaling in the antioxidant function of PIC.

## Discussion

Accumulating evidence has established that plant polyphenols such as curcumin, resveratrol, ferulic acid, ellagic acid, quercetin, chlorogenic acid, and catechin exhibit potent antioxidant properties and therapeutic potential against oxidative stress and its related pathologies [20, 21]. Particularly resveratrol has attracted extensive attention owing to its beneficial effects on human and animal health [22–24]. As a resveratrol analog, PIC features two hydroxyl groups in ortho position of the resveratrol structure. PIC exhibits an 11-fold higher peroxyl radical-scavenging activity than its parent compound, resveratrol [25]. In piglets, dietary PIC supplementation mitigated hepatic injury, likely via the modulation of redox homeostasis, maintenance of mitochondrial function, and prevention of excessive apoptosis [26]. However, information on the supplementation effects of PIC in weaning piglets and its potential mechanism remained insufficient.

The growth performance of piglets is intrinsically associated with their health status, with oxidative stress typically leading to impaired growth performance [1, 27]. Many multifunctional natural compounds exert health-promoting effects, improving growth and production performance, in livestock animals [28, 29]. Jia et al. [26] studied the effects of PIC administration on weaning piglet performance under short-term acute stress induced by diquat. They found no significant differences during the first week post-weaning, but demonstrated that PIC restored the body weight reduction induced by diquat challenge. However, this study did not evaluate the effects of PIC on weaned piglet growth performance throughout the nursery period. In the present investigation, the administration of medium and high doses of PIC significantly improved the ADG during the early weaning phase and throughout the nursery period, and high-dose PIC significantly improved the feed conversion efficiency. While we did not use an in vivo oxidative stress challenge model, our study clearly demonstrated the growth-promoting effects of PIC in weaning piglets. Further studies are needed to confirm its beneficial effects, particularly under oxidative stress conditions, in piglets.

Weaning induces multiple stress pathways in piglets, disrupting intestinal redox homeostasis and leading to oxidative stress [30]. Early weaning compromises intestinal antioxidant capacity in piglets, as evidenced by elevated MDA and ROS levels coupled with significantly reduced antioxidant enzyme activities in the jejunum [20]. PIC has been demonstrated to stimulate the antioxidant system and enhance antioxidant enzyme capacity, thereby mitigating oxidative stress damage in various cell lines and hosts. Treatment with PIC conferred protection against oxidative stress in skeletal muscle cells through upregulation of antioxidant enzymes, including HO1 and SOD1 [13]. PIC administration improved hepatic SOD activity and reduced lipid peroxidation in lipopolysaccharide-induced endotoxemic mice [14]. In the study in piglets by Jia et al. [26], dietary PIC administration improved hepatic SOD and GPX activities and decreased the amount of MDA after diquat challenge. In porcine intestinal epithelial cells, PIC pretreatment significantly attenuated deoxynivalenol-induced oxidative damage through coordinated upregulation of protein expression of phase II detoxification enzymes (NQO1 and GCLM) and transcriptional activation of antioxidant genes (*CAT*, *SOD1*, *PRX3*, and *GSTA4*) [31]. In the present study, PIC supplementation improved piglet serum T-AOC and CAT activity and decreased the amount of MDA, indicating its antioxidant potential in piglets. In vitro, it enhanced antioxidative gene expression and enzyme activity. To our knowledge, this represents the first experimental demonstration of the antioxidant efficacy of PIC in piglets through feeding trials and intestinal epithelial cell studies. Our results provide novel insights supporting the application of PIC as a functional additive in pig production.

Early weaning reduces gut microbial diversity and induces microbiota dysbiosis, enhancing the susceptibility to diarrheal diseases and other health complications, in piglets [32]. Intestinal oxidative stress during the weaning stage triggers gut microbiota dysbiosis, thereby contributes to the pathogenesis of post-weaning diarrhea and enteric infections. The present study demonstrated that dietary PIC supplementation alters the gut microbiota composition, which may contribute to the maintenance of intestinal homeostasis. *Blautia* is a functional genus with probiotic characteristics [33]. In weaning piglets supplemented with a composite probiotic, *Blautia* abundance was significantly negatively correlated with MDA contents, and positively with GSH activity [34]. In line herewith, significant negative correlation between *Blautia* abundance and MDA levels was observed in our study. Increased *Faecalibacterium* abundance has been found to benefit intestinal antioxidant capacity and gut barrier integrity [35]. Consistent herewith, we found a significant positive correlation between *Faecalibacterium* abundance and T-AOC, and negative correlation with MDA levels, in weaning piglets. *Blautia* and *Faecalibacterium* were the dominant bacteria in the medium-dose supplementation group in the present study, which indicates that PIC-induced elevation of these bacteria contributes to enhanced systemic antioxidant capacity. In drinking water supplemented with acidifier, Xu et al. [36] found a significant positive correlation between *Subdoligranulum* abundance and serum antioxidant capacity. In the present study, *Subdoligranulum* was the dominant bacterium in the high-dose supplementation group. We found a negative correlation between *Subdoligranulum* relative abundance and serum MDA concentrations and positive associations with T-AOC and CAT activity (although statistical significance was not reached), implying that increased *Subdoligranulum* abundance may promote antioxidant defenses. *Norank_o_RF39* has been proposed as a potentially harmful bacterium and may be associated with reduced antioxidant enzyme activity [37]. In line herewith, we found negative correlations between *norank_o_RF39* and T-AOC and CAT activity, supporting its potential negative impact on host antioxidant function. Collectively, our results indicate that PIC positively modulates the intestinal microbial community, including enhancing the abundance of bacteria with antioxidant activity, thus enhancing the antioxidant capacity and growth rate of piglets.

Phosphomethylpyrimidine synthase plays an essential role in the biosynthesis of thiamine (vitamin B_1_) [38], which is pivotal in sulfur metabolism and hence antioxidant capacity [39]. KEGG pathway enrichment analysis revealed that phosphomethylpyrimidine synthase [EC 4.1.99.17] was significantly enriched by PIC supplementation, suggesting the potential benefit of PIC in improving piglet antioxidant status. Cobalamin has been shown to promote porcine oocyte maturation while reducing ROS generation and elevating GSH levels [40]. Adenosylcobinamide kinase [EC 2.7.1.156], a key enzyme in cobalamin biosynthesis [41], was upregulated upon PIC supplementation and thus may have contributed to the enhanced antioxidant capacity in piglets. Finally, PIC supplementation significantly enriched methionine synthase [EC 2.1.1.13], which participates in methionine biosynthesis. In swine, dietary supplementation with 0.12% dl-methionine analogs significantly increased serum T-SOD activity and GSH content while improving growth performance [42].

The in vivo results presented above demonstrated the antioxidant effect of PIC on weaning piglets. To further elucidate the underlying mechanism, we established an H_2_O_2_-induced oxidative-injury model in IPEC-J2 cells to evaluate the antioxidant capacity of PIC and its potential pathways. PIC's oxidative stress-mitigating effects were comprehensively assessed using in silico, in vitro, and mouse models [43]. H_2_O_2_ can directly enter cells and induce reversible oxidative stress; therefore, it is widely employed as an oxidative stress inducer in in vitro systems [44]. In IPEC-J2 cells, H_2_O_2_ treatment stimulated MDA secretion and triggered an intracellular ROS burst. Pretreatment with PIC significantly suppressed both MDA production and H_2_O_2_-induced ROS accumulation. These findings align with previous findings that PIC pretreatment effectively inhibits ROS and MDA accumulation in oxidative stress-damaged cells and animal models, ameliorating oxidative stress-induced cellular damage [45, 46]. Apoptosis is typically triggered when cells experience oxidative stress. Oxidative stress can trigger apoptosis through the coordinated upregulation of pro-apoptotic factors and downregulation of anti-apoptotic mediators at both transcriptional and translational levels [47]. PIC downregulated Bcl-XL and inhibited the activation of caspase-3 and caspase-8 in PC12 cells incubated with hydrogen peroxide or SIN-1, thus inhibiting apoptosis [48]. PIC supplementation significantly attenuated H_2_O_2_-induced apoptosis in ARPE-19 cells, as evidenced by a reduced Bax/Bcl-2 ratio and decreased cleavage of caspase-3 and PARP, potentially through the modulation of Akt phosphorylation status [45]. In our study, PIC alleviated H_2_O_2_-induced oxidative damage and apoptosis in porcine epithelial cells. However, the signaling pathways involved, and whether PIC affects mitochondrial or non-mitochondrial apoptotic pathways, require further investigation.

Nrf2 is a crucial redox-sensitive transcription factor that serves as the primary regulator of certain antioxidant enzymes and detoxification genes, playing a pivotal role in maintaining cellular redox homeostasis [49]. Key antioxidant factors regulated by Nrf2 include NQO1, HO1, SOD, CAT, and related enzymes [50]. PIC protected retinal pigment epithelium cells against H_2_O_2_-mediated damage via Nrf2 signaling and *GCLc*, *SOD*, and *HO1* antioxidant gene transcription [45], indicating that PIC may exert its cytoprotective effects through Nrf2 pathway activation. Our data showed that PIC pretreatment potently activated Nrf2 signaling, as evidenced by significantly elevated Nrf2 phosphorylation levels as compared to those in control and H_2_O_2_ treatments. This activation was accompanied by increased antioxidant enzyme expression, indicating that Nrf2 transcriptional activity mediates the cytoprotective effects of PIC against H_2_O_2_-induced oxidative damage. To validate Nrf2 pathway involvement, we assessed MDA production and ROS accumulation following *Nrf2* knockdown in IPEC-J2 cells. Notably, the protective effects of PIC against H_2_O_2_-induced cellular damage were completely abolished under Nrf2-deficient conditions, confirming that Nrf2 signaling is essential for PIC-mediated cytoprotection against oxidative stress in porcine intestinal epithelial cells. Our results indicated that PIC may promotes phosphorylation-dependent nuclear translocation of Nrf2, which then binds to the antioxidant response element and upregulates the transcription of downstream antioxidant enzyme genes, thereby decreasing the levels of stress products (MDA, ROS and 8-OHDG) and effectively attenuating oxidative injury. PIC’s antioxidant effects may also involve other signaling pathways, such as PI3K/Akt or STAT3, or organelle functional regulation [45, 51]. More comprehensive investigations are warranted to fully elucidate the precise molecular mechanisms underlying the antioxidant properties of PIC on porcine cells.

## Conclusions

This study provides the first experimental evidence for the beneficial effects of dietary PIC supplementation on weaning piglet growth performance, demonstrating that its mechanism may involve gut microbiota modulation and Nrf2-mediated antioxidant defense system activation. These findings highlight PIC acts as a potential antioxidant agent, thereby enhancing growth performance and health status in piglets. The optimal level of PIC supplementation to the diet of weaning piglets was found to be 200 mg/kg. Further investigations are warranted to fully elucidate its mechanisms of action, its application in appropriate animal models, and its bioavailability and toxicity in animals to investigate its efficacy as a natural antioxidant in livestock production.

## Supplementary Information

Supplementary Material 1: Table S1. Primer sequences used for quantitative real-time PCR.

Supplementary Material 2: Table S2. Antibodies used in western blotting.

Supplementary Material 3. Protein marker and original gels of the Western blots in the manuscript.

## Data Availability

The data generated and/or analyzed during the current study are available from the corresponding authors upon request.

## Abbreviations

ADFI: Average daily feed intake
ADG: Average daily gain
BAX: Bcl-2-associated X protein
BCL2: B-cell lymphoma 2
CASP3: Caspase 3
CAT: Catalase
GAPDH: Glyceraldehyde-3-phosphate dehydrogenase
GSH: Glutathione
HO1: Heme oxygenase-1
LDH: Lactate dehydrogenase
MDA: Malondialdehyde
NQO1: Quinone oxidoreductase-1
Nrf2: Nuclear factor erythroid 2-related factor 2
ROS: Reactive oxygen species
T-AOC: Total antioxidant capacity
T-SOD: Total superoxide dismutase
8-OHDG: 8-Hydroxy-2′-deoxyguanine
