# Supplementary material for: Piceatannol enhances antioxidant capacity and growth in weaned piglets by regulating of Nrf2-mediated redox homeostasis and modulating of the related gut microbiota
Source: J Anim Sci Biotechnol. 2026 Feb 1;17:17. doi: 10.1186/s40104-025-01320-8 (PMC12861067; doi:10.1186/s40104-025-01320-8)
Supplement: Supplementary file 1 — Supplementary Material 1: Table S1. Primer sequences used for quantitative real-time PCR. [file 40104_2025_1320_MOESM1_ESM.docx]

**Table S1** Primer sequences used for quantitative real-time PCR

| **Gene^1^** | **Primer sequences (5'→3')** | **Accession number** | **Product length, bp** |
| --- | --- | --- | --- |
| *GAPDH* | F：CCTGTTGCTGTAGCCAAATTC  R：GCTACACTGAGGACCAGGTTG | XM_021091114.1 | 146 |
| *SOD1* | F：GCAGGTCCTCACTTCAATCC  R：CTTCCAGCATTTCCCGTCTT | NM_001190422 | 248 |
| *CAT* | F：CACACATACCCATTCGTCACT  R：CAGCCCTAACCTTCACTTACC | NM_214301 | 157 |
| *GPX1* | F：CGATGCCACTGCCCTCAT  R：GGCCCACCAGGAACTTCTC | NM_214201 | 98 |
| *HO1* | F：GTTTGAGGAGGTGCAGGAGC  R：GAGTGTCAGGACCCATCGGA | NM_001004027 | 184 |
| *NQO1* | F：AGTATCCTGCCGAGACTGCTCTG  R：CACAAGGTCTGCGGCTTCCAC | NM_001159613 | 95 |
| *BAX* | F：AAGCGCATTGGAGATGAACT  R：CGATCTCGAAGGAAGTCCAG | XM_013998624.2 | 251 |
| *BCL2* | F：TGTGTGGAGAGCGTCAACCG  R：CCCATACAGCTCCACAAAGGCAT | XM_021099593.1 | 138 |
| *Nrf2* | F: CCCATTACAAAAAGACAAACATTC  R: GCTTTTGCCCTTAGCTCATCTC | XM_013984303 | 72 |

^1^*GAPDH* Glyceraldehyde-3-phosphate dehydrogenase, *SOD1* Superoxide dismutase 1, *CAT* Catalase, *GPX1* Glutathione peroxidase 1, *HO-1* Heme oxygenase-1, *NQO1* NAD(P)H: quinone oxidoreductase 1, *BAX* BCL2 associated X protein, *BCL2* B-cell lymphoma-2, *Nrf2* = nuclear factor-erythroid2-related factor 2
