# Supplementary material for: Piceatannol enhances antioxidant capacity and growth in weaned piglets by regulating of Nrf2-mediated redox homeostasis and modulating of the related gut microbiota
Source: J Anim Sci Biotechnol. 2026 Feb 1;17:17. doi: 10.1186/s40104-025-01320-8 (PMC12861067; doi:10.1186/s40104-025-01320-8)
Supplement: Supplementary file 2 — Supplementary Material 2: Table S2. Antibodies used in western blotting. [file 40104_2025_1320_MOESM2_ESM.docx]

**Table S2** Antibodies used in western blotting

| **Antibody^1^** | **Source** | **Manufacturers** | **Cat#** |
| --- | --- | --- | --- |
| β-actin | Rabbit | Bioss Biotechnology, Beijing, China | bs-0061R |
| Bax | Rabbit | Affinity Biosciences, Melbourne, Victoria, Australia | AF0120 |
| Bcl2 | Mouse | Bioss Biotechnology, Beijing, China | bsm-33411M |
| Cleaved Caspase3 | Rabbit | Affinity Biosciences, Melbourne, Victoria, Australia | AF7022 |
| Nrf2 | Rabbit | Abmart, Shanghai, China | T55136 |
| p-Nrf2 | Rabbit | Bioss Biotechnology, Beijing, China | bs-2013R |

^1^*β-actin* Beta-actin, *Bax* BCL2 associated X protein, *Bcl2* B-cell lymphoma-2, *Cleaved Caspase3* Cleaved cysteinyl aspartate specific proteinase 3, *Nrf2* Nuclear factor-erythroid2-related factor 2, *p-Nrf2* Phosphorylated nuclear factor-erythroid2-related factor 2
