# Supplementary material for: Piceatannol enhances antioxidant capacity and growth in weaned piglets by regulating of Nrf2-mediated redox homeostasis and modulating of the related gut microbiota
Source: J Anim Sci Biotechnol. 2026 Feb 1;17:17. doi: 10.1186/s40104-025-01320-8 (PMC12861067; doi:10.1186/s40104-025-01320-8)
Supplement: Supplementary file 3 — Supplementary Material 3. Protein marker and original gels of the Western blots in the manuscript. [file 40104_2025_1320_MOESM3_ESM.docx]

**Piceatannol enhances antioxidant capacity and growth in weaned piglets by regulating of Nrf2-mediated redox homeostasis and modulating of the related gut microbiota**

The protein markers [#MP102 (10-180 kDa), purchased from Vazyme Biotech Co., Ltd. (Nanjing, China)] used are shown below:

**
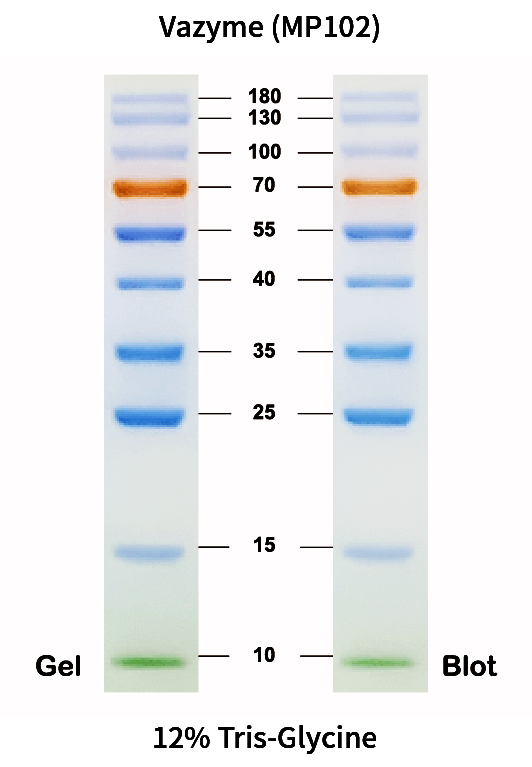
**

**Protein marker and original gels of the Western blots in the manuscript:**

**Fig. 4** PIC attenuates apoptosis in H_2_O_2_-induced IPEC-J2 cells

**Fig. 4B** Bcl2


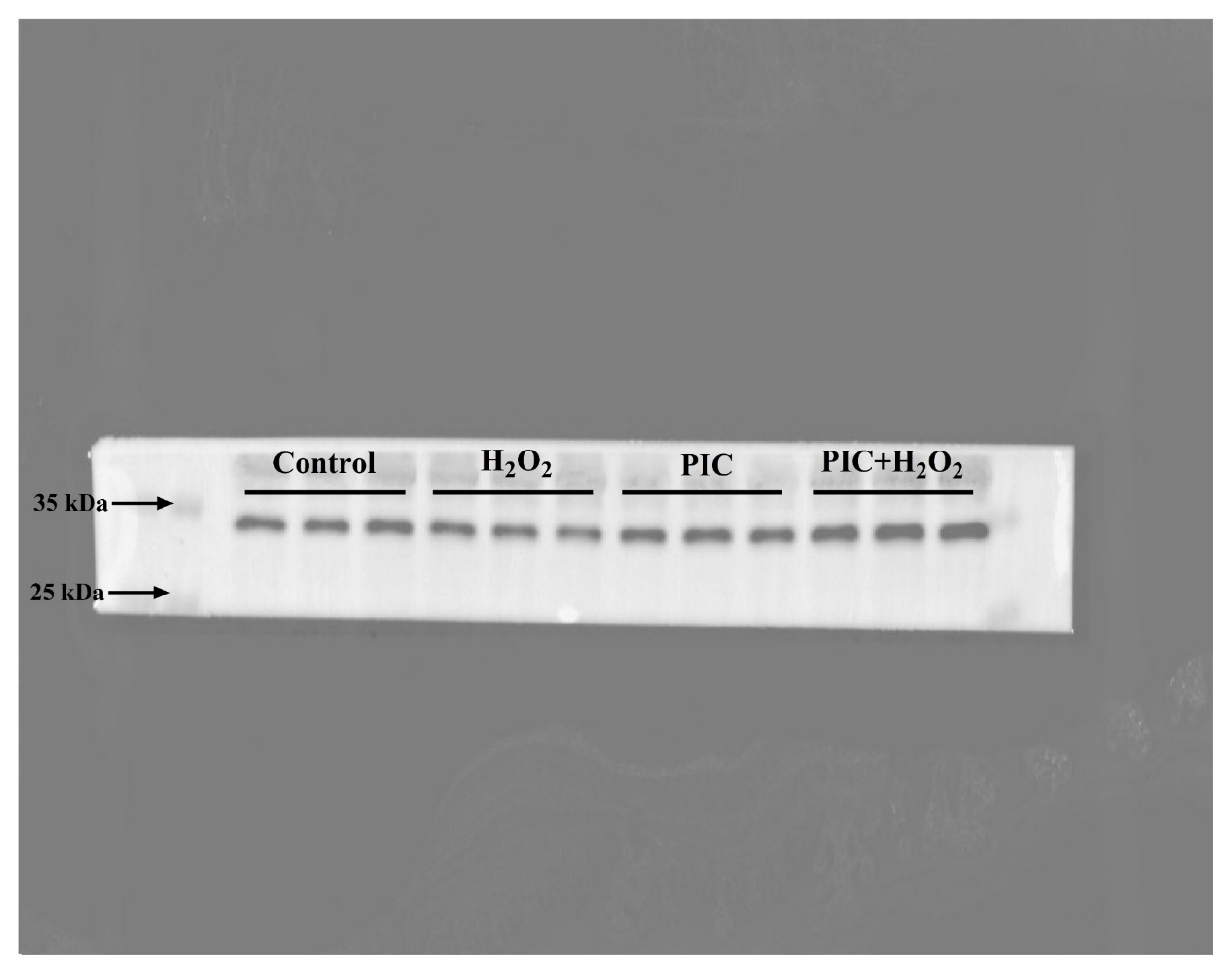


Control: Bcl2 from control-treated IPEC-J2; H_2_O_2_: Bcl2 from H_2_O_2_-treated IPEC-J2; PIC: Bcl2 from PIC-treated IPEC-J2; PIC+H_2_O_2_: Bcl2 from PIC+H_2_O_2_-treated IPEC-J2

**Fig. 4B** Bax


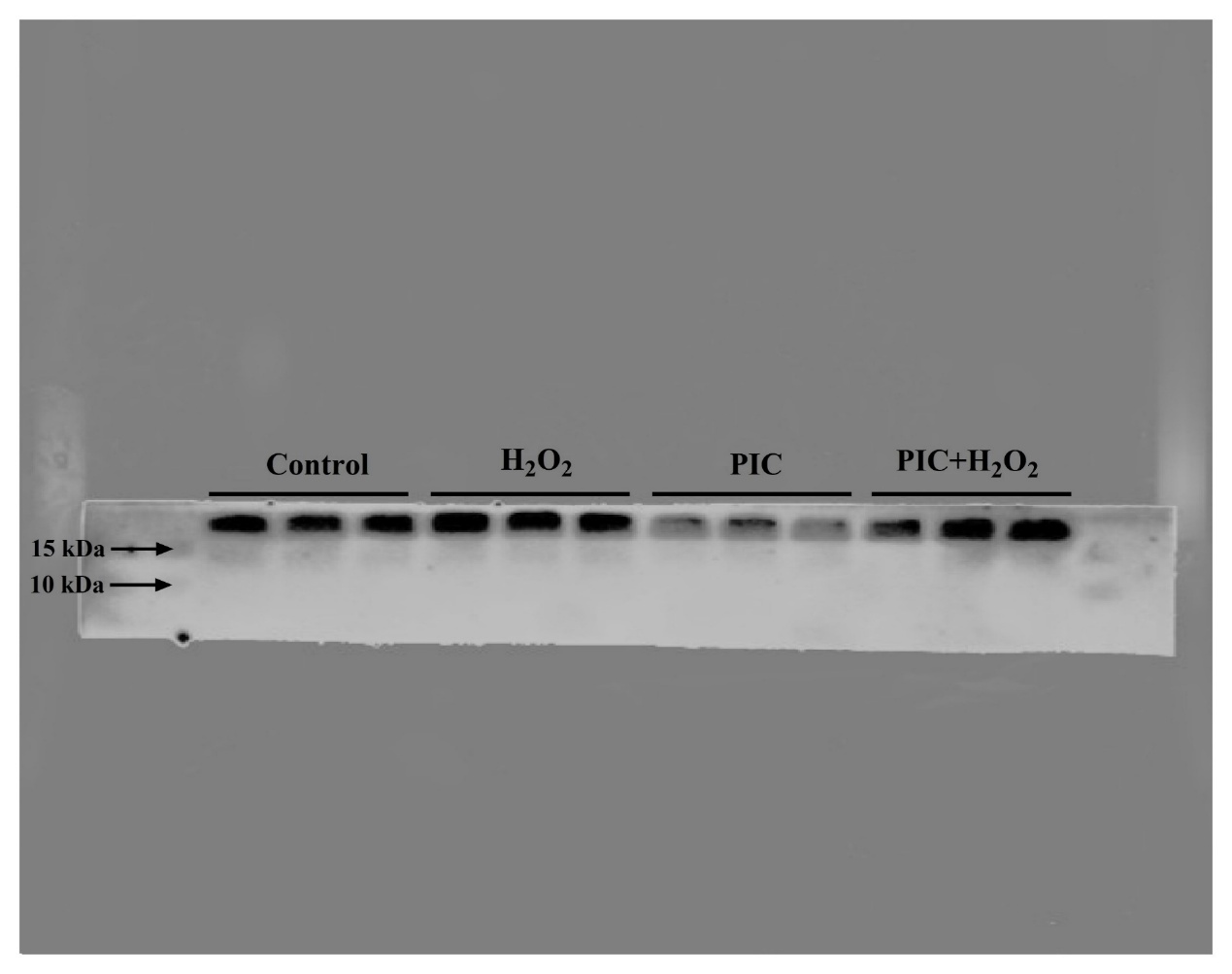


Control: Bax from control-treated IPEC-J2; H_2_O_2_: Bax from H_2_O_2_-treated IPEC-J2; PIC: Bax from PIC-treated IPEC-J2; PIC+H_2_O_2_: Bax from PIC+H_2_O_2_-treated IPEC-J2

**Fig. 4B** Cleaved Caspase3


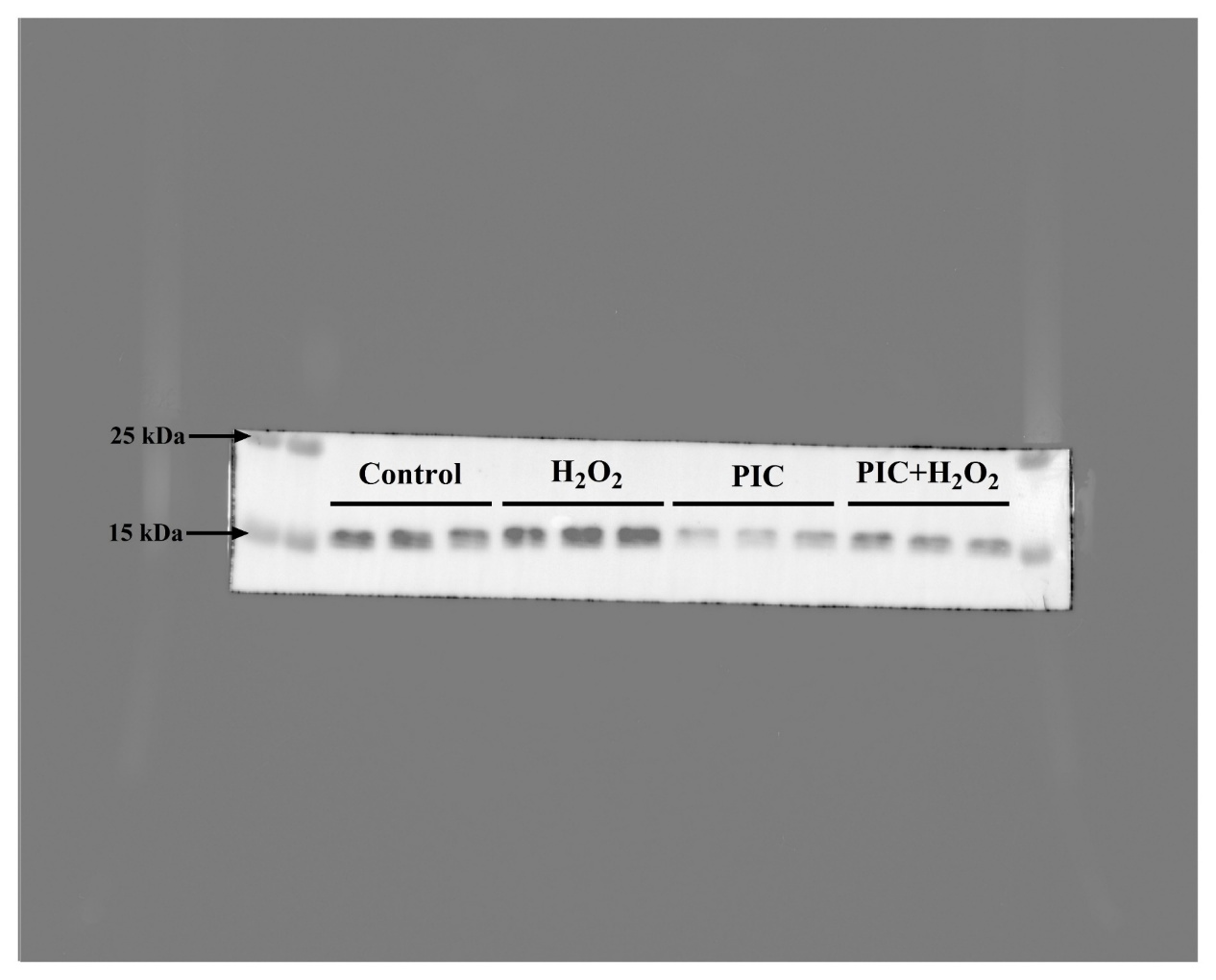


Control: Cleaved Caspase3 from control-treated IPEC-J2; H_2_O_2_: Cleaved Caspase3 from H_2_O_2_-treated IPEC-J2; PIC: Cleaved Caspase3 from PIC-treated IPEC-J2; PIC+H_2_O_2_: Cleaved Caspase3 from PIC+H_2_O_2_-treated IPEC-J2

**Fig. 4B** β-actin


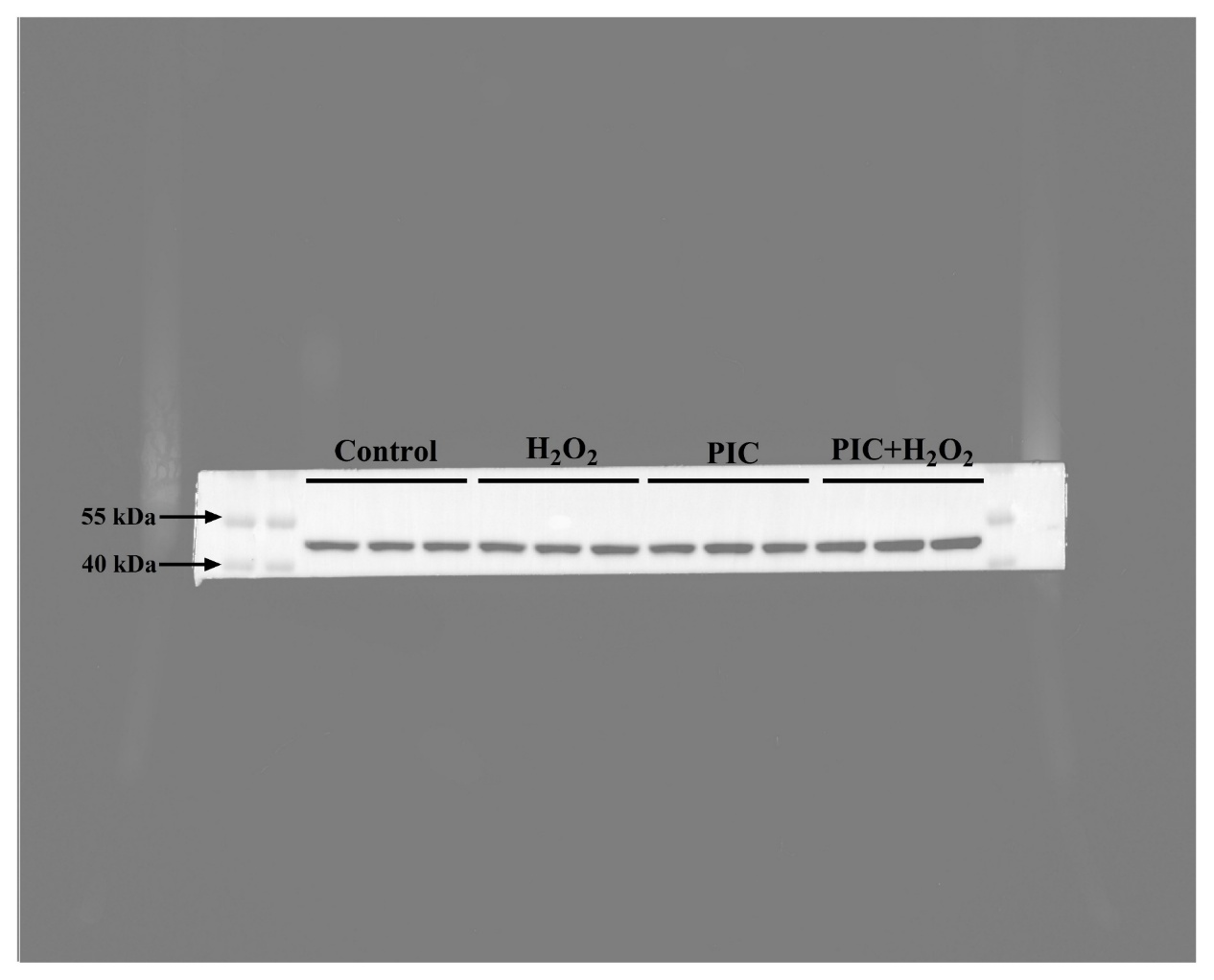


Control: β-actin from control-treated IPEC-J2; H_2_O_2_: β-actin from H_2_O_2_-treated IPEC-J2; PIC: β-actin from PIC-treated IPEC-J2; PIC+H_2_O_2_: β-actin from PIC+H_2_O_2_-treated IPEC-J2

**Fig. 6** PIC alleviates oxidative stress via the Nrf2 pathway in H_2_O_2_-induced IPEC-J2 cells

**Fig. 6A** p-Nrf2


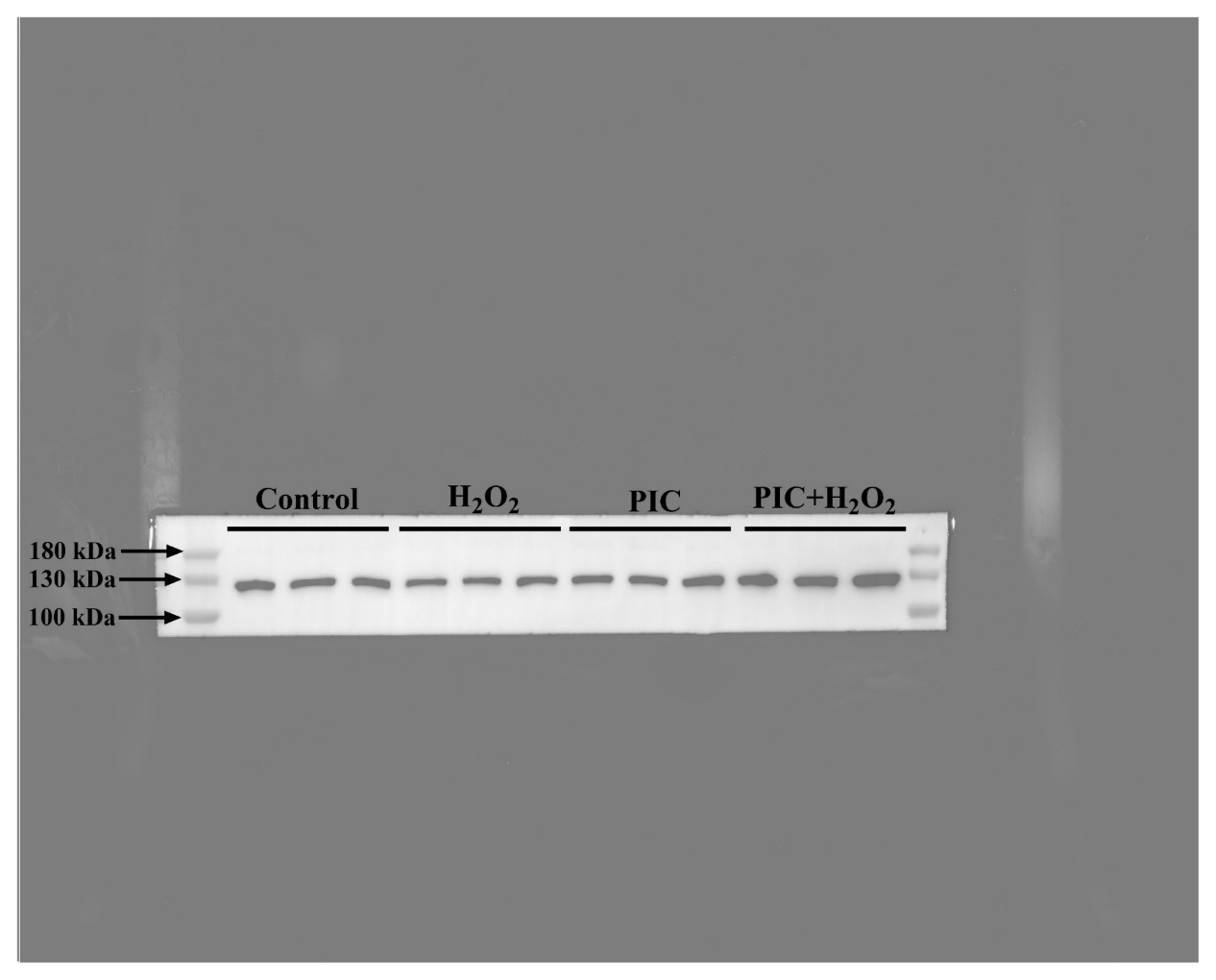


Control: p-Nrf2 from control-treated IPEC-J2; H_2_O_2_: p-Nrf2 from H_2_O_2_-treated IPEC-J2; PIC: p-Nrf2 from PIC-treated IPEC-J2; PIC+H_2_O_2_: p-Nrf2 from PIC+H_2_O_2_-treated IPEC-J2

**Fig. 6A** Nrf2


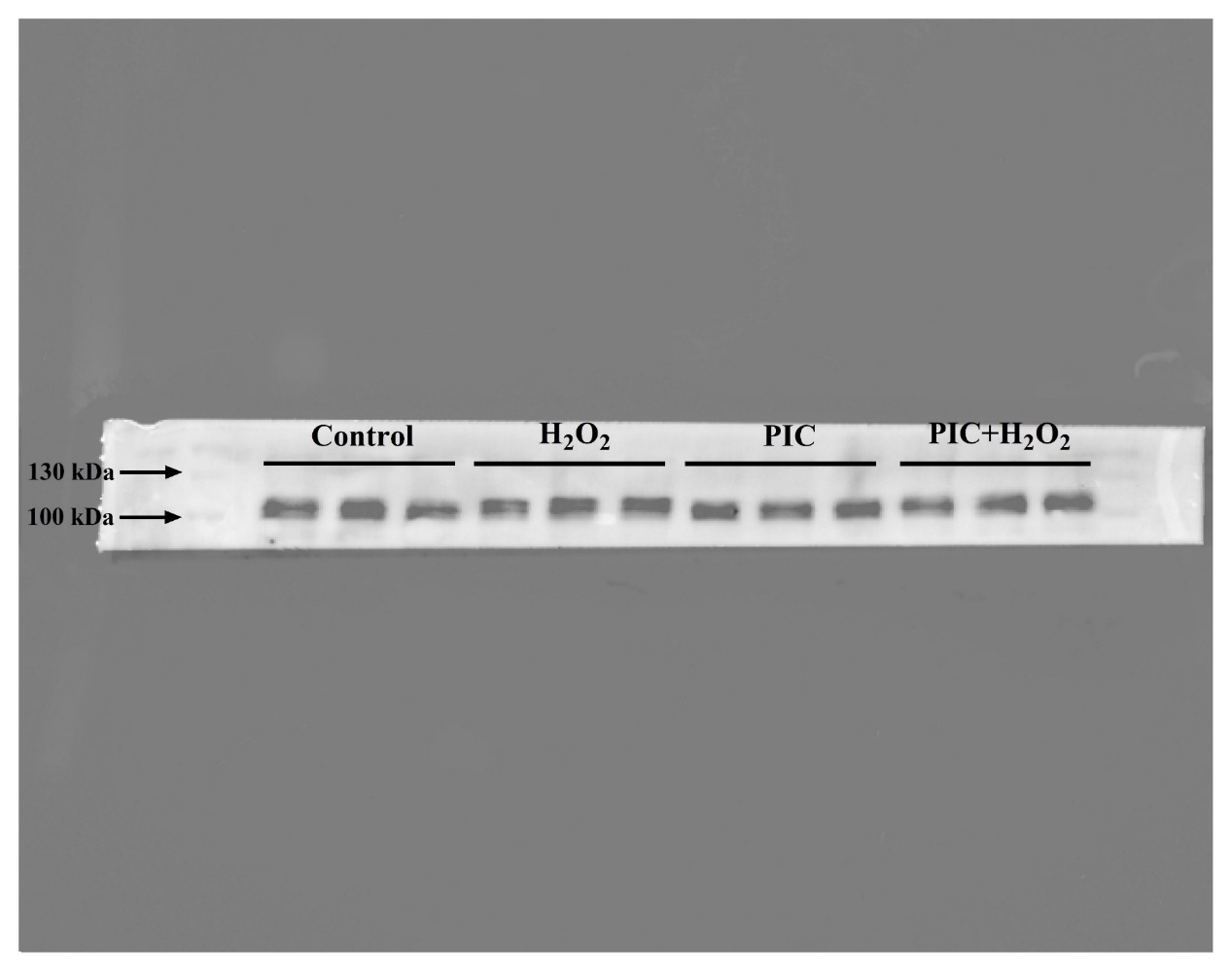


Control: Nrf2 from control-treated IPEC-J2; H_2_O_2_: Nrf2 from H_2_O_2_-treated IPEC-J2; PIC: Nrf2 from PIC-treated IPEC-J2; PIC+H_2_O_2_: Nrf2 from PIC+H_2_O_2_-treated IPEC-J2

**Fig. 6A** β-actin


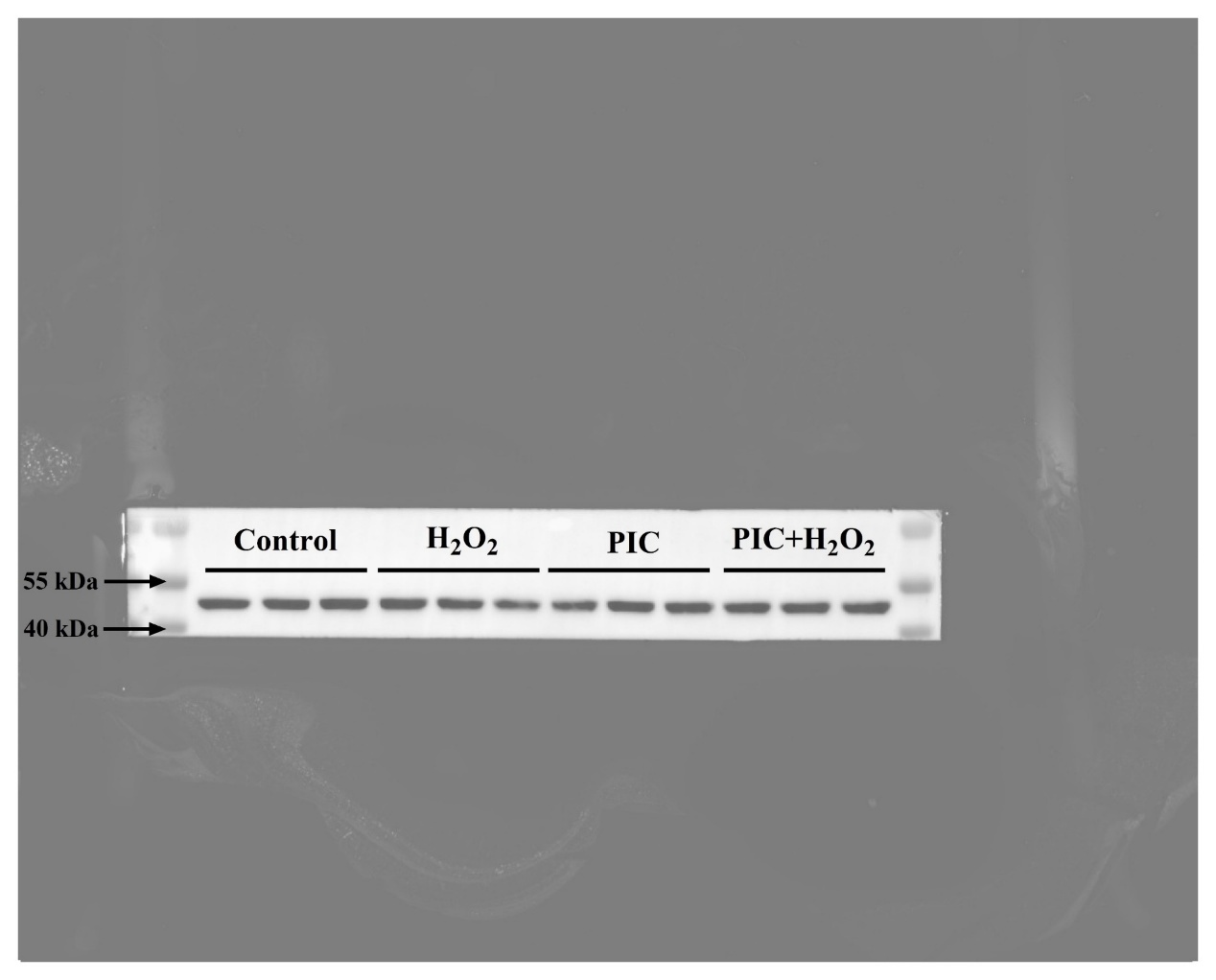


Control: β-actin from control-treated IPEC-J2; H_2_O_2_: β-actin from H_2_O_2_-treated IPEC-J2; PIC: β-actin from PIC-treated IPEC-J2; PIC+H_2_O_2_: β-actin from PIC+H_2_O_2_-treated IPEC-J2

**Fig. 6C** Nrf2


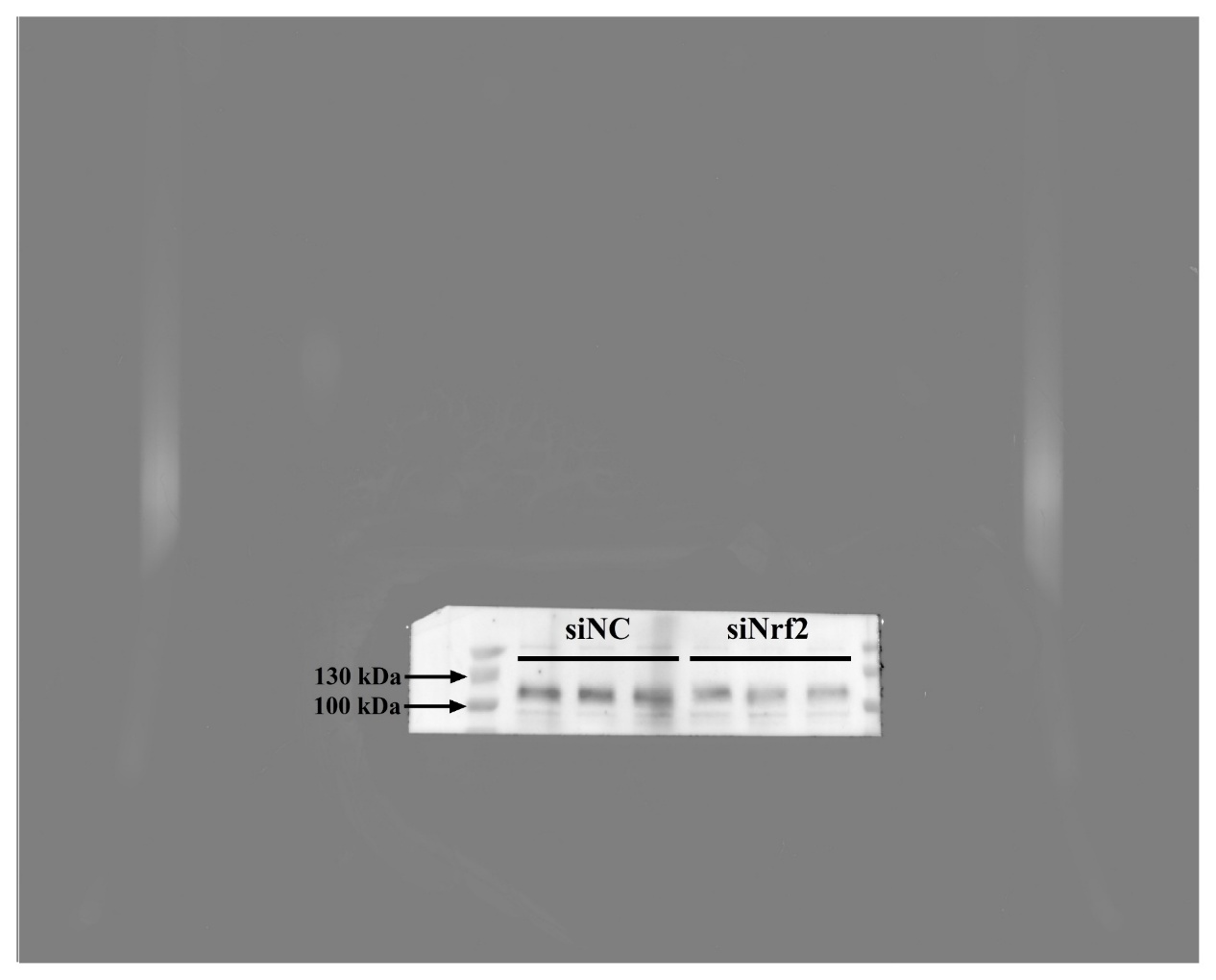


siNC: Nrf2 from siNC-treated IPEC-J2; siNrf2: Nrf2 from siNrf2-treated IPEC-J2

**Fig. 6C** β-actin


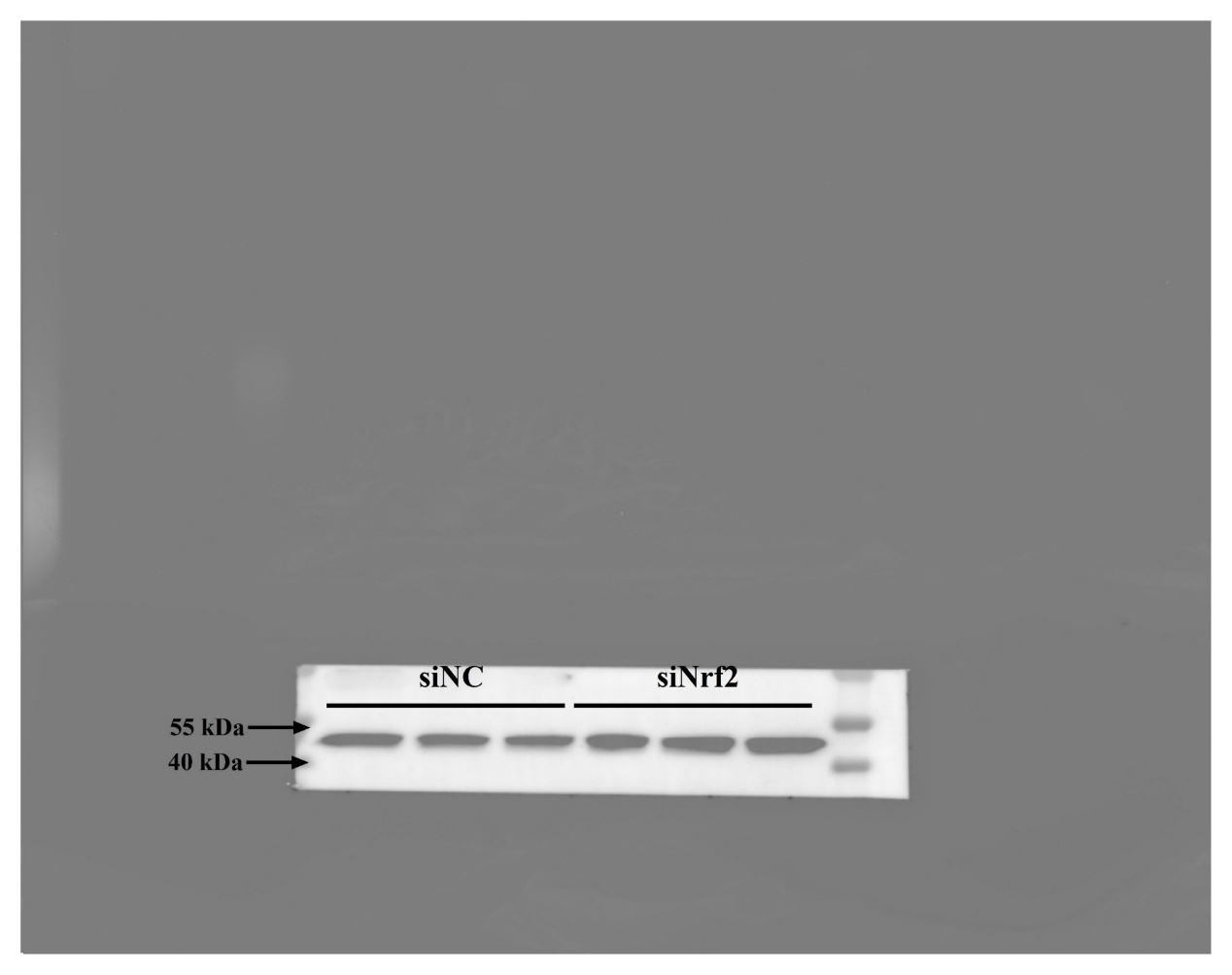


siNC: β-actin from siNC-treated IPEC-J2; siNrf2: β-actin from siNrf2-treated IPEC-J2
